# Protectin DX resolves fracture-induced postoperative pain in mice via neuronal signaling and GPR37-activated macrophage efferocytosis

**DOI:** 10.1172/JCI190754

**Published:** 2026-01-16

**Authors:** Yize Li, Sangsu Bang, Jasmine Ji, Jing Xu, Min Lee, Sharat Chandra, Charles N. Serhan, Ru-Rong Ji

**Affiliations:** 1 Center for Translational Pain Medicine, Department of Anesthesiology, Duke University Medical Center, Durham, North Carolina, USA.; 2 Center for Experimental Therapeutics and Reperfusion Injury, Department of Anesthesiology, Perioperative and Pain Medicine, Brigham and Women’s Hospital, Harvard Medical School, Boston, Massachusetts, USA.; 3 Department of Dermatology,; 4 Department of Cell Biology,; 5 Department of Neurobiology, and; 6 Department of Integrative Immunobiology, Duke University Medical Center, Durham, North Carolina, USA.

**Keywords:** Cell biology, Neuroscience, Therapeutics, G protein-coupled receptors, Macrophages, Pain

## Abstract

Protectin DX (PDX) is a member of the superfamily of specialized proresolving mediators and exerts anti-inflammatory actions in animal models; however, its signaling mechanism remains unclear. Here, we demonstrate the analgesic actions of PDX in a mouse model of tibial fracture–induced postoperative pain (fPOP). Intravenous early- and late-phase treatment of PDX (100 ng/mouse) effectively alleviated fPOP. Compared with protectin D1 (PD1)/neuroprotectin D1, DHA, steroids, and meloxicam, PDX provided superior pain relief. While dexamethasone and meloxicam prolonged fPOP, PDX shortened the pain duration. The analgesic effects of PDX were abrogated in *Gpr37*^−/−^ mice, which displayed deficits in fPOP resolution. PDX was shown to bind GPR37 and induce calcium responses in peritoneal macrophages. LC-MS/MS–based lipidomic analysis revealed that endogenous PDX levels were approximately 10-fold higher than those of PD1 in muscle at the fracture site. PDX promoted macrophage polarization via GPR37-dependent phagocytosis and efferocytosis through calcium signaling in vitro, and it further enhanced macrophage viability and efferocytosis in vivo via GPR37. Finally, PDX rapidly modulated nociceptor neuron responses by suppressing C-fiber–induced muscle reflex in vivo and calcium responses in DRG neurons ex vivo and by reducing TRPA1/TRPV1-induced acute pain and neurogenic inflammation in vivo. Our findings highlight multiple benefits of PDX to manage postoperative pain and promote perioperative recovery.

## Abstract 2

In this study, Li et al. demonstrated that protectin DX can resolve postoperative pain via GPR37 activation, macrophage efferocytosis, and neuronal signaling in mice with bone fracture.

## Graphical abstract

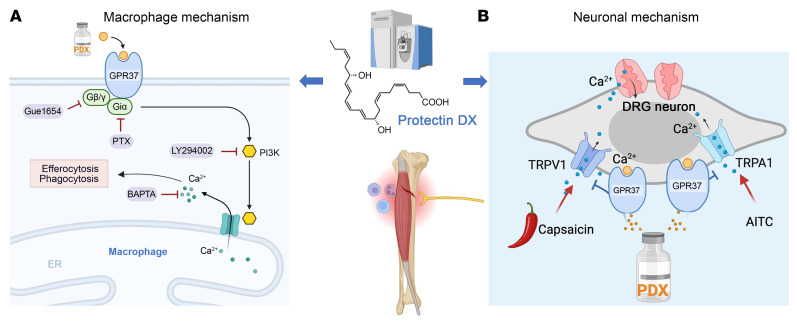

## Introduction

Fracture-related postoperative pain (fPOP) is associated with tissue injury in joints and muscles, local inflammation, as well as nerve injury and neuroinflammation in the peripheral and central nervous systems (1, 2). Although multimodal analgesia is widely used, postoperative pain after orthopedic surgery is still frequently undertreated, hindering recovery (3). Opioids have traditionally been the mainstay for postoperative pain management. However, concerns over their side effects, such as the potential for abuse and respiratory suppression, which drive the ongoing opioid epidemic (4), have created an urgent need to optimize nonopioid analgesic options (3, 5). Furthermore, anti-inflammatory treatments such as steroids and NSAIDs produce gastrointestinal complications and may further impair the resolution of inflammatory pain and low-back pain (6, 7). Thus, there is an urgent need to develop more effective and safe pain therapeutics.

Proresolution pharmacology was proposed for controlling inflammation and pain (8) based on the potent analgesic actions of resolvins (1–300 ng/mouse) via different administration routes in animal models of pain (9). Specialized proresolving mediators (SPMs) consist of omega-3 eicosapentaenoic acid–derived E-series resolvins, docosahexaenoic acid–derived (DHA-derived) D-series resolvins, protectins and maresins, cysteinyl-SPMs, as well as omega-6 arachidonic acid–derived lipoxins (10). DHA is crucial for brain development (11), and DHA-derived SPMs consist of resolvin D1–D6 (RvD1–RvD6), maresin 1 and 2 (MaR1 and MaR2), and protectin D1 (PD1) (12, 13). Given its critical role in neuroprotection, PD1 is also termed neuroprotectin D1 (NPD1) (14). SPMs, produced during the resolution phase of inflammation, bind to specific GPCRs and regulate the activities of different cell types, including neutrophils, macrophages, T cells, epithelial cells, and neurons, promoting inflammation resolution, tissue repair, and pain relief (15, 16) (Supplemental Figure 1A; supplemental material available online with this article; https://doi.org/10.1172/JCI190754DS1). Mechanistically, SPMs resolve inflammation via macrophage phagocytosis and efferocytosis (17). PD1/NPD1 was shown to resolve inflammatory pain and infection-induced pain through GPR37-mediated macrophage phagocytosis (18, 19). Notably, pain resolution or chronicity is associated with SPM production (20, 21).

Protectin DX (PDX) was identified as a stereoisomer of PD1 in 2006 (22) and was further chemically characterized in 2009 (23). Compared with PD1, PDX has been shown to exhibit different chemical and biological properties (24). For example, PDX (10S,17S-diHDHA) is biosynthesized via double dioxygenation, as determined using molecular oxygen (^18^O) incorporation in PDX and mass spectrometry, and it was shown to reduce neutrophil infiltration in mouse peritonitis in vivo, albeit less potently than PD1 (22) (Supplemental Figure 1B). PDX alleviates insulin resistance and type 2 diabetes (25) and inhibits blood platelet activity at higher concentrations (micromolar range) (23, 26). However, key issues remain to be resolved regarding the therapeutic potential of PDX in pain management: the receptor for PDX is unknown; endogenous PD1 and PDX levels in health and disease have not been characterized; optimal pain models, delivery routes, and dosing for PDX remain undefined, with SPM analgesic efficacy varying by model (20, 27) and sex (28); and the immune-neuronal mechanisms of PDX are largely unexplored. To this end, we tested the inhibitory actions of PDX on the development, maintenance, and recovery of fPOP and further investigated the molecular and cellular mechanisms using a clinically relevant orthopedic surgery pain model (29). Our findings demonstrate that intravenously administered PDX, at low doses (30–100 ng/mouse), can potently mitigate fPOP and promote the resolution of postoperative pain via GPR37.

## Results

### Early perisurgical treatment of PDX mitigates the development of fPOP.

PDX and PD1/NPD1 were obtained from Cayman Chemical and validated using UV spectroscopy and mass spectrometry, matching the UV spectra, retention times, and fragmentation patterns of the compounds to those of known standards (Supplemental Figures 2 and 3). Before testing lipid mediators or NSAIDs, we first investigated the time course of postoperative pain in male and female CD1 mice after sham surgery and tibial fracture surgery. Mice with sham surgery exhibited no changes in mechanical pain (paw withdrawal threshold [PWT] in von Frey tests, Supplemental Figure 4A), cold pain (acetone test, Supplemental Figure 4B), and heat pain (Hargreaves tests, Supplemental Figure 4C). However, following tibial fracture surgery, animals exhibited increased mechanical, cold, and heat pain on the first day, and these evoked pains persisted for 14 days and fully recovered by 21 days after surgery (Supplemental Figure 4, A–C). We also examined spontaneous pain by assessing guarding behavior, which appeared on the first day after surgery, maintained on day 7, but returned to baseline on day 10 (Supplemental Figure 4D). The grimace test showed spontaneous pain only on days 3 and 5 after fracture (Supplemental Figure 4E).

**Figure 1:**
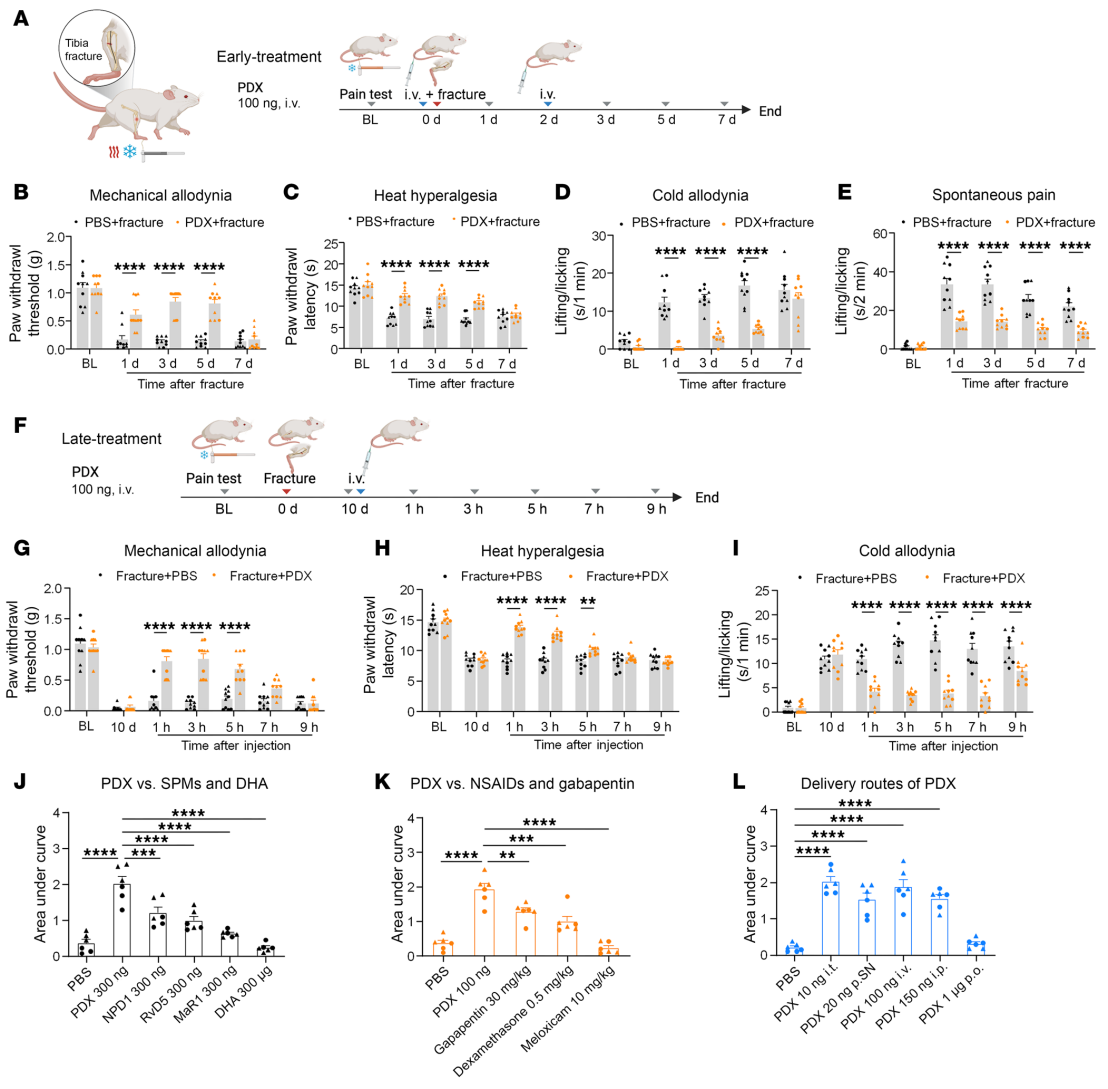
Perisurgical pre- and posttreatment PDX attenuates tibial fPOP in CD1 mice. (**A**) Schematic of the tibial fracture model and PDX pretreatment timeline. CD1 mice received 2 i.v. injections of PDX (100 ng, 100 μL) or PBS (100 μL): 1 immediately before fracture surgery and 1 after fracture day 2. BL, baseline. (**B**–**E**) Effects of PDX and vehicle on fPOP measured on postsurgical days 1, 3, 5, and 7, including mechanical pain (PWT by von Frey test; **B**), thermal pain (paw withdrawal latency by Hargreaves test; **C**), cold pain (duration of pain with lifting/licking behavior in acetone test; **D**), and spontaneous pain (duration of lifting/licking behavior; **E**). (**F**) Posttreatment paradigm. In a separate cohort, mice received a single i.v. injection of PDX (100 ng, 100 μL) or PBS on postfracture day 10. (**G**–**I**) Posttreatment PDX also reduced mechanical (**G**), thermal (**H**), and cold (**I**) pain. (**J** and **K**) Comparison of PDX with other SPMs (NPD1, RvD5, and MaR1; 300 ng/mouse) and DHA (300 μg/mouse; **J**), as well as anti-inflammatory drugs (dexamethasone, 0.5 mg/kg; meloxicam, 10 mg/kg) and gabapentin (30 mg/kg; **K**). (**L**) Route-of-administration comparison showing effective analgesia following i.t. (10 ng), p.SN. (20 ng), i.v. (100 ng), i.p. (150 ng), or p.o. (1 μg) delivery of PDX. Data in **J**–**L** are presented as AUC of PWT. Data are presented as mean ± SEM. Statistics: 2-way ANOVA with Bonferroni’s post hoc test (**B**–**E** and **G**–**I**) and 1-way ANOVA with Tukey’s post hoc test (**J**–**L**). **P < 0.01, ***P < 0.001, ****P < 0.0001; *n* = 10 (**B**–**E** and **G**–**I**), *n* = 6 (**J**–**L**); equal number of male and female mice; triangles, male; circles, female.

We next investigated the effects of PDX early treatment on fPOP, administered during the perisurgical period, with the first injection given right before surgery and the second injection given 2 days after surgery (Figure 1A). We performed an intravenous dose–response study (1–100 ng per mouse), a standard perioperative route (30). The von Frey testing showed no effects on PWT at 1–10 ng, a transient effect at 30 ng, and robust antinociception lasting for 5 h at 100 ng (Supplemental Figure 5A); thus, a 100 ng dose was used for most experiments in this study. Compared with PD1/NPD1, 300 ng NPD1 was required to achieve a similar effect to 100 ng PDX (Supplemental Figure 5, A and B). ED_50_ analysis (52 ng vs. 205 ng) confirmed an approximately 4-fold greater potency of PDX over NPD1 (Supplemental Figure 5C).

We next evaluated early PDX treatment in fPOP during the first postoperative week across mechanical, thermal, cold, and spontaneous pain (Figure 1A). Intravenous PDX (100 ng) markedly reduced mechanical allodynia, thermal hyperalgesia, cold allodynia, and spontaneous pain on days 1, 3, and 5 and continued to suppress spontaneous pain on day 7 (all *P* < 0.0001; Figure 1, B–E), with no sex differences (Supplemental Figure 5D). Thus, early i.v. PDX effectively attenuates acute fPOP.

### Late PDX treatment alleviates persistent fPOP and outperforms standard analgesics.

Because fPOP persists for 2–3 weeks, we tested whether delayed PDX administration (100 ng, i.v., day 10) reduces established pain in male and female CD1 mice (Figure 1F). PDX significantly suppressed mechanical, thermal, and cold hypersensitivity at 1–5 h (all *P* < 0.0001 to *P* < 0.01) and produced prolonged relief of cold pain up to 9 h (Figure 1, G–I), with no sex differences (Supplemental Figure 5E). We next compared PDX with DHA, MaR1, NPD1, and RvD5 (all at 100 pmol, i.v.). PDX produced the greatest analgesic effect, with a significantly larger AUC than all comparators (*P* < 0.001; Figure 1J and Supplemental Figure 6A). Postoperative pain is typically treated using dexamethasone (steroid), meloxicam (NSAID), and gabapentin (31). Notably, PDX (100 ng; ~0.003 mg/kg) also outperformed gabapentin (30 mg/kg, i.p.) and dexamethasone (0.5 mg/kg, i.v.), despite 100- to 5,000-fold lower doses; meloxicam (10 mg/kg, i.v.) was ineffective in late-phase fPOP (Figure 1K and Supplemental Figure 6B). Finally, PDX showed robust efficacy via intrathecal (10 ng), perisciatic (20 ng), intraperitoneal (150 ng), and i.v. delivery, each producing approximately 5 h of analgesia (Figure 1L and Supplemental Figure 7). Oral gavage, even at 1,000 ng, yielded only a transient benefit. Together, these data demonstrate that delayed PDX treatment effectively reverses established fPOP, exhibits superior potency to other SPMs and clinical analgesics, and is effective across multiple delivery routes.

### PDX and anti-inflammatory treatments differentially regulate the duration of fPOP.

**Figure 2:**
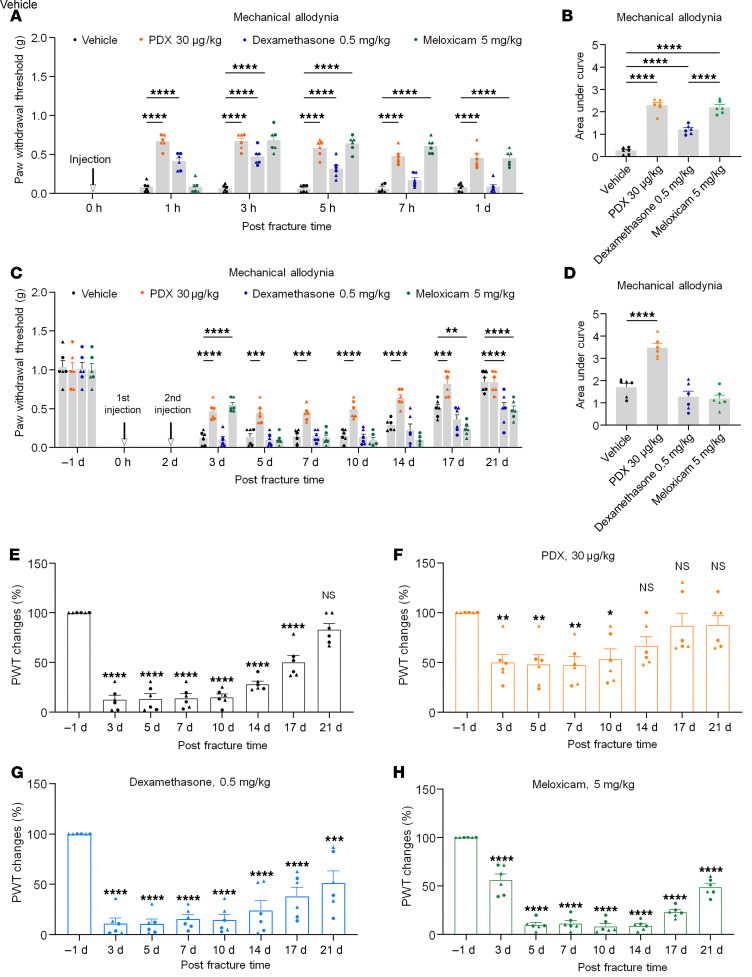
Effects of PDX, dexamethasone, and meloxicam on acute fPOP and the resolution of fPOP in CD1 mice. (**A** and **B**) Acute effects after the first i.v. injection of PDX (30 μg/kg), dexamethasone (0.5 mg/kg), meloxicam (5 mg/kg), or vehicle (PBS, 100 μL) given immediately before the fracture surgery. The data are shown as PWT (**A**) and AUC of PWT (**B**). (**C** and **D**) Sustained effects of the drugs on the resolution of fPOP following the second injection given 48 h after tibial surgery. The data are shown as PWT (**C**) and AUC of PWT (**D**). (**E**–**H**) Time course of fPOP showing distinct recovery of fPOP following different pain treatments. The data are shown as percentage of preinjury baseline PWT after the second injection of vehicle (**E**), PDX (**F**), dexamethasone (**G**), and meloxicam (**H**). Data are presented as mean ± SEM. Statistics: 2-way ANOVA with Bonferroni’s post hoc test (**A** and **C**) and 1-way ANOVA with Bonferroni’s post hoc test (**B**, **D**, and **E**–**H**). *P < 0. 05, **P < 0. 01, ***P < 0.001, ****P < 0.0001; *n* = 6; equal number of male and female mice; triangles, male; circles, female.

Anti-inflammatory drugs such as steroids and NSAIDs may delay inflammatory pain resolution (6, 8). To compare their effects with PDX, we administered PDX (100 ng/mouse), dexamethasone (0.5 mg/kg), or meloxicam (5 mg/kg) immediately before surgery (Figure 2A). Dexamethasone produced transient analgesia (1–5 h), meloxicam showed delayed but sustained effects (3–24 h), and PDX elicited both rapid and lasting relief (1–24 h, *P* < 0.0001). AUC analysis indicated stronger acute antinociception with PDX and meloxicam than dexamethasone (Figure 2B). To assess longer-term benefit, a second dose was given at 48 h (Figure 2C). Dexamethasone provided no sustained benefit and worsened pain at day 21; meloxicam produced transient relief but later increased pain (days 14–21). In contrast, PDX significantly improved pain from days 3 to 17 (*P* < 0.001), as confirmed by AUC analysis (Figure 2D).

Recovery profiles further showed full resolution by day 21 in vehicle controls (Figure 2E), accelerated recovery by day 14 with PDX (Figure 2F), and delayed or absent recovery by day 21 with dexamethasone or meloxicam (Figure 2, G and H). Thus, unlike current anti-inflammatory treatments that can delay pain resolution, PDX both relieves and accelerates recovery from fPOP.

### PDX is endogenously produced in muscle tissue at the fracture site.

**Figure 3:**
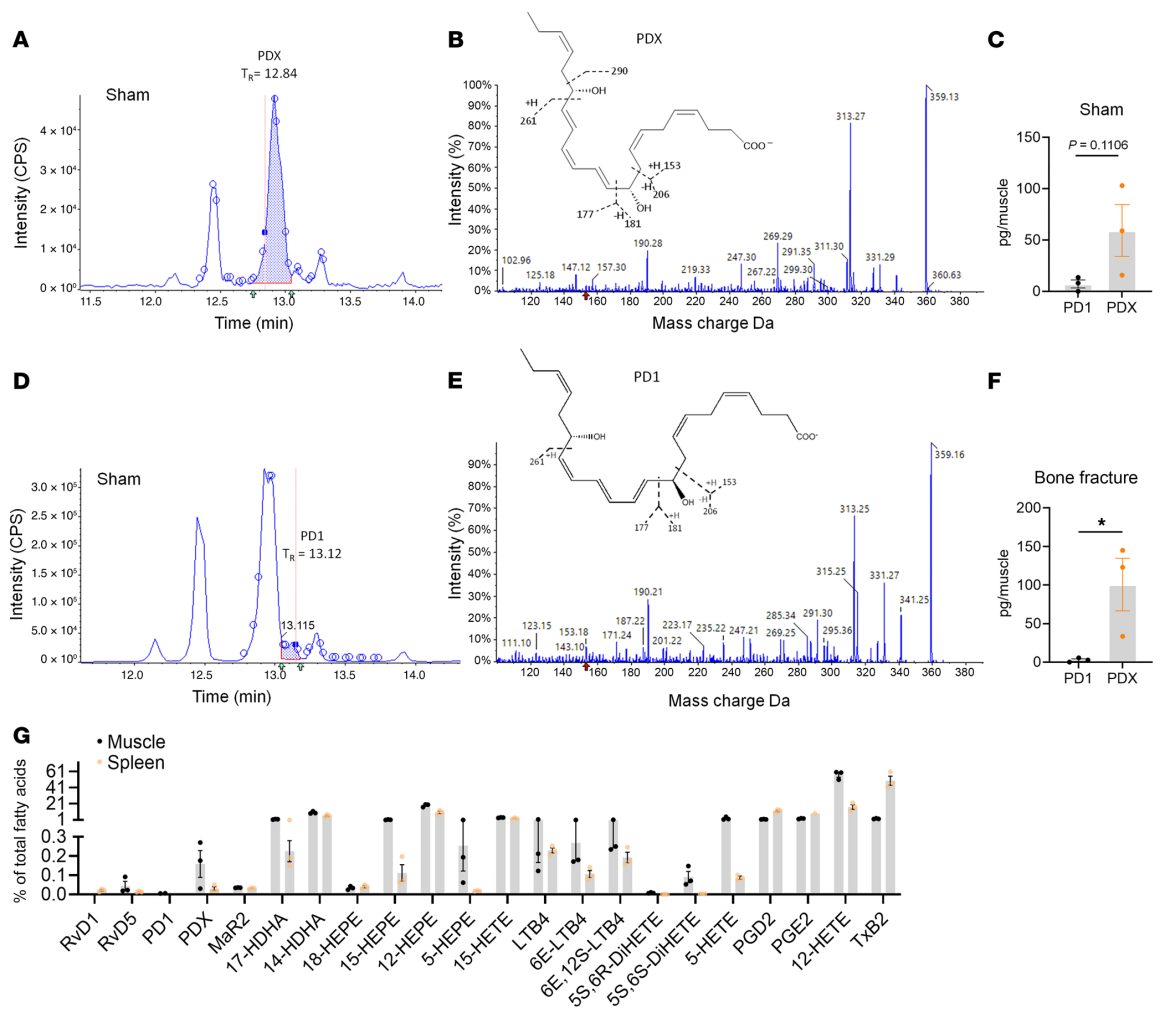
Lipidomic analysis reveals distinct production of PDX and PD1 at the fracture site of CD1 mice. (**A** and **D**) Chromatogram showing retention times for PDX (**A**) and PD1 (**D**) in bone fracture or sham muscle tissues. CPS, counts per second. (**B** and **E**) Mass spectra showing fragmentation pattern as well as prominent ions and chemical structure of PDX (**B**) and PD1 (**E**). (**C** and **F**) Quantification of PDX and PD1 levels in sham muscle tissue (**C**; *n* = 3) or bone fracture muscle tissue (**F**; *n* = 3). (**G**) Normalized lipid mediator levels as percentage of total fatty acid level in muscle and spleen (*n* = 3) tissues of mice with bone fracture. Data are presented as mean ± SEM. Statistics: unpaired *t* test (**C** and **F**). *P < 0.05. CD1 male mice were given either sham surgery or tibial bone fracture surgery, and muscle and spleen tissues were collected 3 days after surgery and stored in PBS. Lipid mediator quantitation was carried out using LC-MS/MS on a SCIEX Triple Quad 7500 system. Lipid mediators were identified by matching retention and prominent ions in their mass spectra to those of authentic results expressed as pg/60 mg spleen tissue.

We performed lipidomic analysis to compare the endogenous production of PD1 and PDX in muscle tissue surrounding the tibial bone (Figure 3, A–F). Lipid mediator quantification was carried out using liquid chromatography and tandem mass spectrometry (LC-MS/MS) on a SCIEX Triple Quad 7500 system. Lipid mediators were identified by matching retention time and prominent ions in their MS/MS to those of in-house authentic lab standards for PDX (Supplemental Figure 2) and PD1 (Supplemental Figure 3). Our results showed the presence of PDX (Figure 3, A and B) and PD1 (Figure 3, D and E) in the muscle tissue. Notably, the endogenous PDX levels were much higher than those of PD1/NPD1 both in naive animals without surgery (Figure 3C, *P* = 0.106) and in animals with bone fracture (*P* < 0.05, Figure 3, C and F, and Supplemental Table 4). Compared with naive animals (<50 pg/sample), PDX was further elevated after bone fracture (near 100 pg/sample, Figure 3, C and F). Strikingly, PDX levels were 10 times greater than PD1 levels in muscle samples (Supplemental Table 4). We also identified greater levels of PDX compared with PD1 in the spleen, an immune organ (Supplemental Table 5). The ratio of PDX in total free fatty acids was also greater in muscle than spleen tissues (Figure 3G). Furthermore, PDX levels were among the highest of all the detected SPMs in muscle and spleen tissues (Supplemental Tables 4 and 5), underscoring its unique role in inflammation resolution and pain modulation.

### PDX binds and activates GPR37 to induce Ca²^+^ signaling.

**Figure 4:**
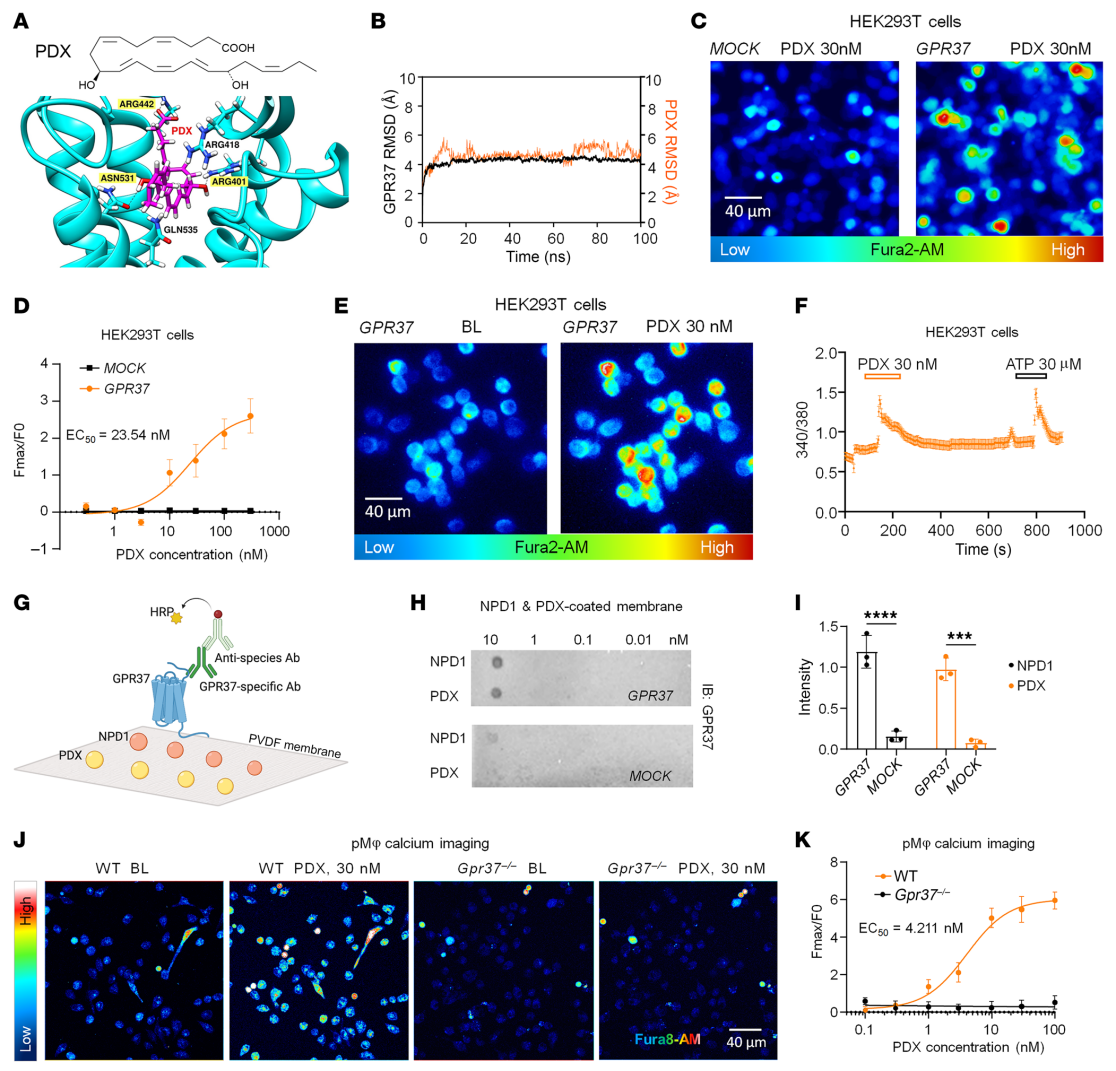
PDX directly binds GPR37 and increases calcium influx in HEK293T cells and pMφs from C57BL/6 mice. (**A**) Structural model of human GPR37 (blue) in complex with PDX (magenta). (**B**) Molecular dynamics simulation showing root mean square deviation (RMSD) of the GPR37–PDX complex (orange) versus GPR37 alone (black) over 100 ns, indicating stable ligand–receptor interaction. (**C**) PDX (30 nM) evoked calcium influx in GPR37-transfected HEK293T cells but not in mock-transfected controls. (**D**) Dose–response curve of PDX-induced calcium signaling in GPR37-expressing HEK293T cells versus mock controls (EC_50_ = 23.54 nM; *n* = 16 reads from 4 cultures). (**E** and **F**) Representative traces (**E**) and quantification (**F**) demonstrate enhanced calcium responses to PDX (30 nM) and ATP (30 μM) in GPR37-expressing HEK293T cells (*n* = 27 cells, 3 cultures). (**G**) Dot blot schematic for assessing binding of GPR37 to PDX and NPD1 using PVDF membranes coated with ligands at graded concentrations. (**H**) Representative blot of PDX and NPD1-coated PVDF membranes incubated with lysates from HEK293T cells with and without GPR37 expression. (**I**) Quantification of dot intensity in HEK293T cell lysates with and without GPR37. *n* = 3 repeats. (**J** and **K**) Calcium imaging in pMφs showing traces (**J**) and quantification (**K**) of calcium responses following PDX (30 nM) treatment in pMφ cultures prepared from WT or *Gpr37*^–/–^. Note that PDX induces dose-dependent responses in WT pMφs but has no effects in *Gpr37*^–/–^ pMφs. EC_50_ of the PDX-induced calcium response is 4.21 nM. *n* = 9 cultures from 3 mice. Data are presented as mean ± SEM. Statistics: 2-way ANOVA with Tukey’s post hoc test (**I**). ***P < 0.001, ****P < 0.0001. Scale bars: 40 μm (**C**, **E**, and **J**).

Because GPR37 serves as a receptor for NPD1 (18, 19) and PDX is structurally related, we examined whether PDX also targets GPR37. Homology modeling and molecular dynamics simulations predicted stable PDX–GPR37 interactions involving ARG442, ASN531, and ARG401, with consistent binding stability over 100 ns (root mean square deviation 4–6 Å; Figure 4, A and B). To test GPR37-mediated signaling, we measured intracellular Ca²^+^ in HEK293T cells expressing human GPR37. PDX (30 nM) evoked robust Ca²^+^ increases only in GPR37^+^ cells (EC_50_ ≈ 23.5 nM), reversible on washout, and ATP produced expected responses (Figure 4, C–F). Lipid overlay assays further confirmed direct binding of PDX and NPD1 to GPR37 (Figure 4, G–I). Native HEK cells showed minimal responses even at 30 nM, consistent with low endogenous GPR37 expression (Supplemental Figure 8, A–C). Given high Gpr37 expression in peritoneal macrophages (19), we evaluated Ca²^+^ responses in WT and *Gpr37*^–/–^ macrophages. PDX triggered rapid, dose-dependent Ca²^+^ elevations in WT macrophages (EC_50_ ≈ 4.2 nM) — notably more sensitive than HEK293T cells — and failed to induce responses in *Gpr37*^–/–^ cells (Figure 4, J and K). Together, these data identify GPR37 as a functional receptor for PDX and demonstrate GPR37-dependent Ca²^+^ signaling in both engineered and native immune cells.

### GPR37 activation accelerates fPOP resolution and mediates PDX-induced pain relief.

**Figure 5:**
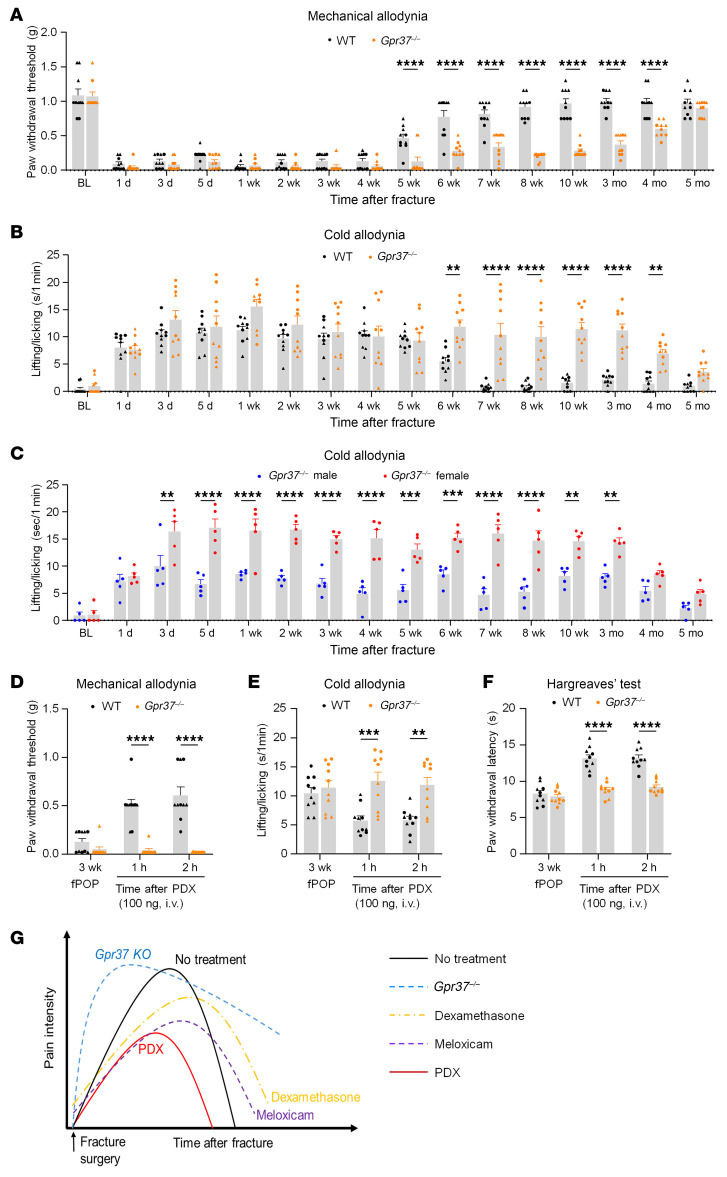
GPR37 mediates PDX-induced pain relief and contributes to the resolution of fPOP in C57BL/6 mice. (**A** and **B**) Time course of mechanical allodynia (von Frey test; **A**) and cold allodynia (acetone test; **B**) following tibial fracture in WT and *Gpr37*^–/–^ mice. *Gpr37*^–/–^ mice showed delayed recovery, indicating impaired resolution of postoperative pain. (**C**) Sex-specific analysis of cold allodynia in *Gpr37*^–/–^ mice reveals persistent cold pain in females up to 3 months after fracture, whereas males exhibit partial recovery. (**D**–**F**) PDX treatment (100 ng, i.v.) reduced fPOP only in WT mice but not in *Gpr37*^–/–^ mice, as assessed by von Frey test (**D**), acetone test (**E**), and Hargreaves test (**F**) in WT and *Gpr37*^–/–^ mice. Data are presented as mean ± SEM. Statistics: 2-way ANOVA with Bonferroni’s post hoc test (**A**–**F**). **P < 0. 01, ***P < 0.001, ****P < 0.0001; *n* = 10 (**A**, **B**, and **D**–**F**), *n* = 5 (**C**); triangles, male; circles, female. (**G**) Schematic illustration of pain resolution following fracture surgery in WT mice under different treatment conditions: (a) no treatment, (b) NSAID (meloxicam), (c) steroid (dexamethasone), (d) PDX treatment, and (e) *Gpr37*^–/–^ mice. Notably, PDX accelerated pain resolution, whereas NSAID and steroid treatments delayed resolution. Also note that *Gpr37*^–/–^ mice failed to resolve pain.

To define the role of GPR37 in postoperative pain, we compared WT and *Gpr37*^−/−^ mice (C57BL/6). In WT mice, mechanical allodynia resolved by approximately 5 weeks and cold hypersensitivity by approximately 6 weeks (Figure 5, A and B), consistent with longer fPOP duration in C57BL/6 than CD1 mice. In contrast, *Gpr37*^−/−^ mice showed markedly prolonged pain, with full recovery only after approximately 5 months and significantly lower PWT from 5 weeks to 4 months (*P* < 0.0001). Thus, GPR37 is essential for fPOP resolution. We also observed sex differences: female *Gpr37*^−/−^ mice exhibited greater cold pain than males from day 3 to month 3 (*P* < 0.01; Figure 5C). To test whether GPR37 mediates PDX action, we administered PDX (100 ng, i.v.) at 3 weeks after fracture. PDX reduced mechanical, thermal, and cold hypersensitivity in WT mice but had no effects in *Gpr37*^−/−^ mice (*P* < 0.01 vs. WT; Figure 5, D–F). These results show that GPR37 promotes resolution of postoperative pain and is required for PDX-induced analgesia.

### Pathway analysis reveals macrophage/neutrophil signaling and wound healing upregulated by PDX treatment in fPOP.

**Figure 6:**
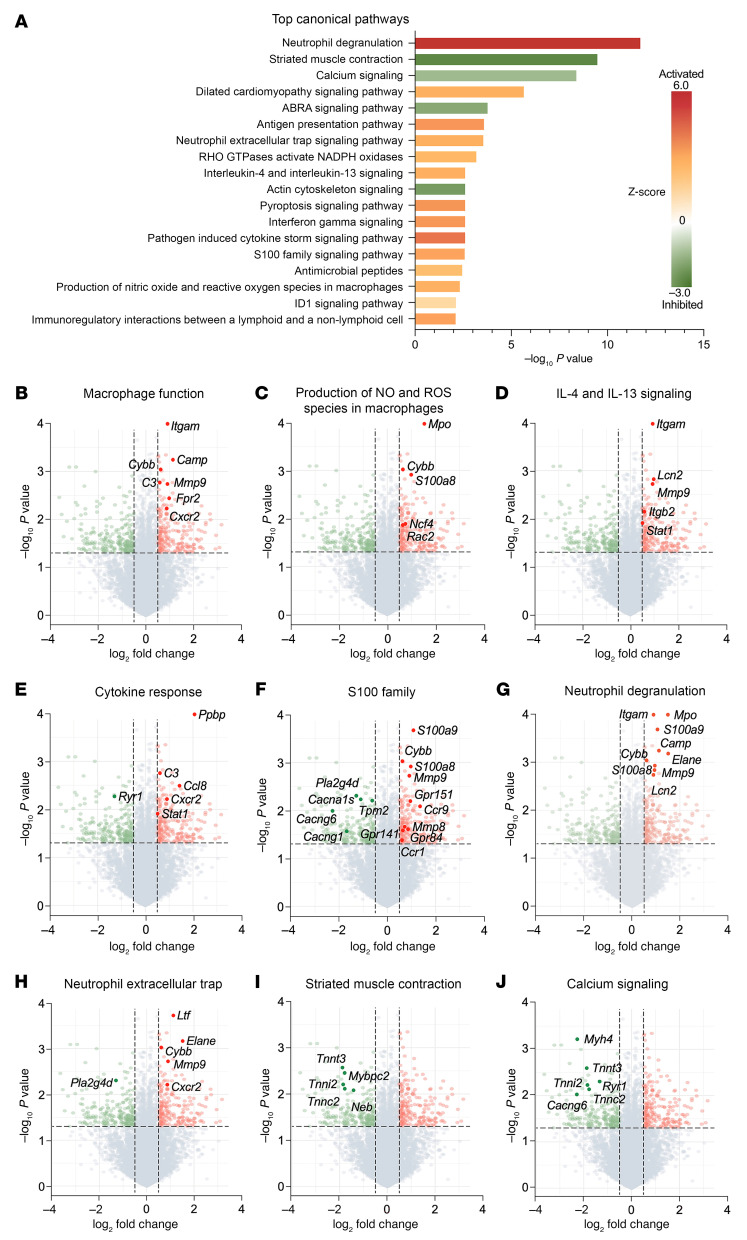
Bulk RNA-seq shows the PDX effects on multiple pathways in DRG of mice with fPOP in CD1 mice. (**A**) Bulk RNA-seq data analysis showing the top canonical pathways in fracture plus PDX versus fracture plus vehicle group. All the pathways show statistical significance of *P* < 0.01 with both upregulations and downregulations, as shown by *z* score. (**B**–**J**) Volcano plots for differentially expressed genes. Red dots denote the upregulated genes, and green dots denote the downregulated genes with > 50% change and *P* < 0.05. The activated pathways are related to macrophage function (**B**), production of NO and ROS species in macrophage genes (**C**), IL-4 and IL-13 signaling genes (**D**), pathogen-induced cytokine genes (**E**), S100 family genes (**F**), neutrophil degranulation genes (**G**), and neutrophil extracellular trap genes (**H**). The inactivated pathways are related to striated muscle contraction genes (**I**) and calcium signaling genes (**J**). *n* = 6 mice (3 males and 3 females) per group.

Primary sensory neurons, glial cells, and immune cells in the dorsal root ganglia (DRG) play an important role in the pathogenesis of pain (32, 33). We collected L3–L5 DRG tissues from mice 3 days after fracture with and without PDX treatment and conducted bulk RNA-seq. The canonical pathway analysis showed significant gene changes related to macrophage and neutrophil function and cytokine responses (Figure 6A). PDX upregulated many genes related to macrophage function, including pathogen recognition (*Camp* and *Cybb*), phagocytosis (*Itgam*), inflammation regulation (*Cxcr2* and *Ccr1*), wound healing (*Ppbp*), and immune response (*C3*), especially S100 family genes (*S100a9* and *S100a8*) and a proresolution gene (*Frp2*) encoding the SPM lipoxin A_4_ receptor ALX/FPR2 (34, 35) (Figure 6, B–H). These results suggest that PDX might trigger an immune activation in the DRG of mice with fPOP, involving macrophages and neutrophils.

### PDX regulates pro- and anti-inflammatory cytokine expression in macrophages via GPR37.

To understand the role of PDX in regulating the expression of key inflammatory cytokines in peritoneal macrophage (pMφ) cultures, we employed qPCR analysis. We cultured pMφs from WT and *Gpr37*^−/−^ mice, treated the cells with PDX (30 nM) and/or LPS (1 μg/mL) for 12 h, and measured the mRNA levels of pro-inflammatory cytokines (*Il1b* and *Tnf*) and anti-inflammatory cytokine *Il10* (Supplemental Figure 9A). In cultured WT pMφs, PDX treatment increased *Il10* mRNA levels and inhibited the LPS-induced increase in *Il1b* and *Tnf* mRNA expression (Supplemental Figure 9B). However, PDX treatment did not reverse the LPS-induced changes in *Il1b*, *Tnf*, and *Il10* in *Gpr37*^−/−^ pMφs (Supplemental Figure 9C). Additionally, ELISA was used to detect the secretion of IL-1β, TNF-α, and IL-10 in the pMφs from WT and *Gpr37*^−/−^ mice (Supplemental Figure 9D). PDX reversed the LPS-induced secretion of pro-inflammatory cytokines (IL-1β and TNF-α) by pMφs. PDX treatment alone also increased the secretion of IL-10 (Supplemental Figure 9, E–G). These effects of PDX were lost in *Gpr37*^−/−^ pMφs (Supplemental Figure 9, H–J). Collectively, our findings suggest an important role of PDX in regulating pro- and anti-inflammatory cytokine expression in macrophages through GPR37.

### PDX accelerates macrophage phagocytosis via GPR37 and Ca^2+^ signaling.

**Figure 7:**
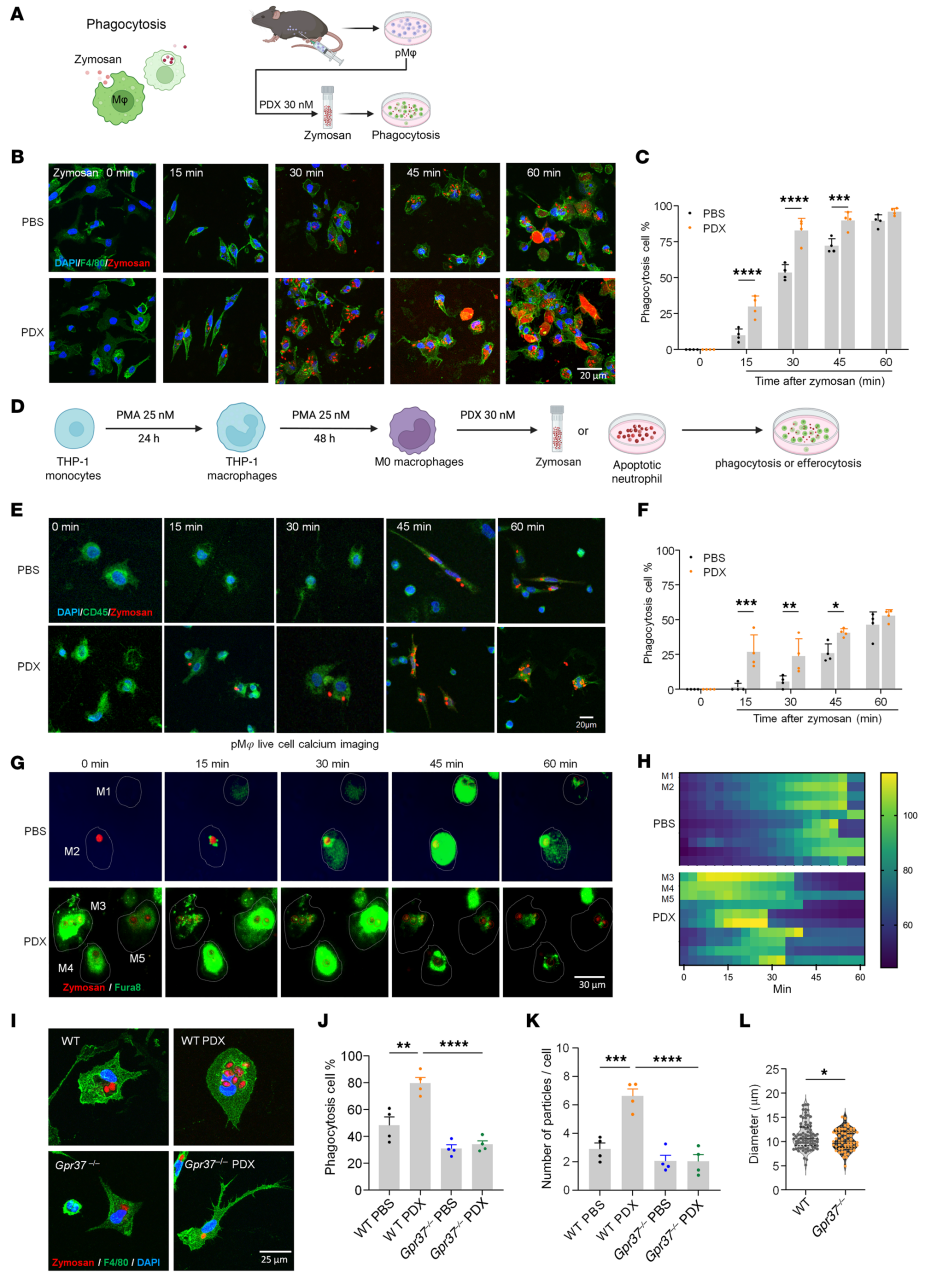
PDX increases macrophage phagocytosis in pMφs of C57BL/6 mice and THP1-derived macrophages via calcium signaling. (**A**) Schematic of zymosan phagocytosis assay in primary pMφs. pHrodo Red zymosan particles (red; activated in acidic phagosomes) were added 1 h before imaging. (**B**) Representative fluorescent images showing time-dependent zymosan uptake in WT pMφs labeled with F4/80 (green) at 0, 15, 30, 45, and 60 min after PBS or PDX treatment. (**C**) Quantification of phagocytic pMφs (% cells containing > 1 particle). (**D**) Schematic of zymosan phagocytosis in THP1 culture. THP1 monocytes were differentiated into macrophages with PMA (25 nM, 48 h). (**E** and **F**) Time-lapse images (**E**) and quantification (**F**) of zymosan uptake in THP1 macrophages (CD45^+^, green) over 60 min. PDX accelerated phagocytosis relative to PBS. (**G**) Calcium imaging of pMφs during phagocytosis, showing increased calcium influx with PDX versus PBS at multiple time points. Fura-8 AM (green) was used for Ca^2+^ detection; zymosan is shown in red. pMφs are indicated by the white dotted outlines and are labeled as M1–M5. (**H**) Heatmap shows time-dependent calcium influx of pMφs after PBS (*n* = 10 cells) or PDX (*n* = 10 cells) incubation, including M1–M5 presented in **G**. This experiment was conducted in 3 cultures. (**I**–**K**) PDX-induced zymosan phagocytosis of pMφs (F4/80^+^, green) from WT and *Gpr37*^–/–^ mice, as shown by images at 0 and 30 min (**I**), and quantification of percentage of positive cells with phagocytosis (**J**) and zymosan particle number in each cell (**K**). *n* = 4 cultures. (**L**) Cell diameters of WT and *Gpr37*^–/–^ pMφs. *n* = 83 cells from 4 cultures from WT mice; *n* = 58 cells from 4 cultures from knockout mice. Data are presented as mean ± SEM. Statistics: 2-way ANOVA with Bonferroni’s post hoc test. *P < 0.05, **P < 0.01, ***P < 0.001, ****P < 0.0001; *n* = 4 cultures/condition (12 mice total, mixed sex; **C** and **D**); *n* = 4 cultures/condition (THP1 cells in **E** and **F**). Scale bars: 20 μm (**B** and **E**), 30 μm (**G**), 25 μm (**I**).

Because NPD1 promotes macrophage phagocytosis (18), we tested whether PDX does the same. pH-sensitive zymosan assays showed that PDX (30 nM, 1 h) significantly accelerated and increased phagocytosis in mouse peritoneal macrophages, with higher phagocytic rates at 15–45 min and near-complete uptake by 60 min (Figure 7, A–C). PDX similarly enhanced phagocytosis in THP1 human macrophages (Figure 7, D–F).

Live Ca²^+^ imaging revealed that PDX advanced intracellular Ca²^+^ elevations coincident with zymosan uptake, whereas vehicle produced delayed Ca²^+^ signals and slower phagocytosis (Figure 7, G and H, and Supplemental Videos 1 and 2), indicating Ca²^+^-dependent phagocytosis. PDX failed to enhance phagocytosis in *Gpr37*^−/−^ macrophages, which also showed reduced baseline uptake and smaller cell size (Figure 7, I–L), demonstrating that GPR37 is required for PDX-driven phagocytosis and macrophage growth.

Mechanistically, PDX-induced phagocytosis was abolished by Ca²^+^ chelation (BAPTA-AM) and significantly inhibited by pertussis toxin, a Gβ/γ blocker, and a PI3K/AKT inhibitor (Supplemental Figure 10B), implicating GPR37–Gi/o–Ca²^+^–PI3K/AKT signaling. Thus, PDX promotes macrophage phagocytosis through GPR37-dependent Ca²^+^ signaling and downstream Gi/βγ/PI3K pathways.

### PDX enhances macrophage efferocytosis via GPR37 and Ca^2+^ signaling.

**Figure 8:**
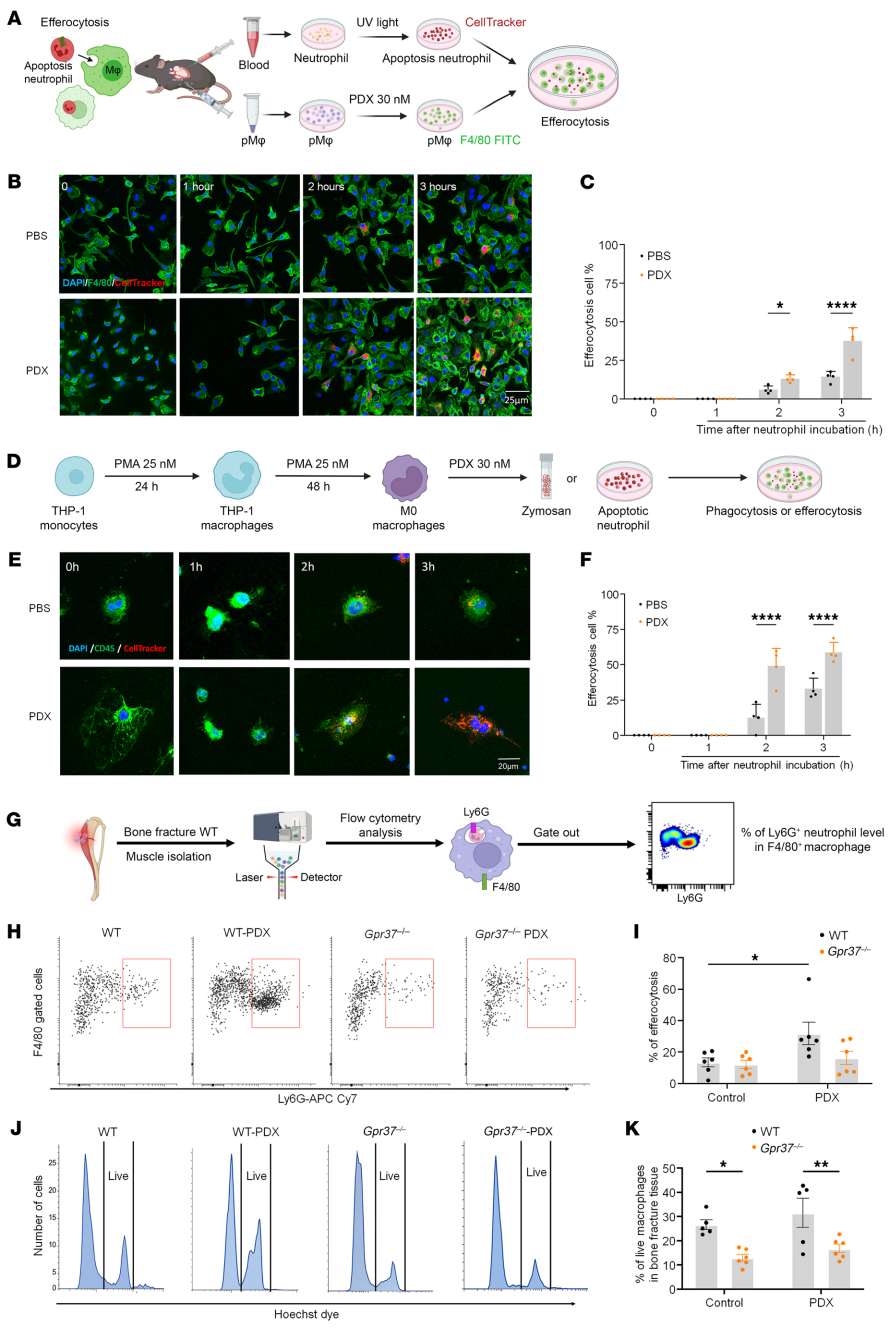
PDX accelerates macrophage efferocytosis of neutrophils in C57BL/6 mice via GPR37. (**A**) Schematic of neutrophil efferocytosis assay in pMφ culture. Neutrophils from mouse whole blood were exposed to UV light for 10 min to induce apoptosis and then labeled with CellTracker (red). The CellTracker-labeled apoptotic neutrophils were incubated with pMφs, which were labeled with F4/80 (green). (**B**) Fluorescent images showing time-dependent efferocytosis in WT pMφs at 0, 1, 2, and 3 h after PBS and PDX treatment. Scale bar: 25 μm. (**C**) Quantification of percentage of pMφs with efferocytosis. The green macrophages containing neutrophils or cell debris were regarded as positive cells. (**D**) Schematic of neutrophil efferocytosis in THP1 culture. PMA (25 nM, 48 h) was used to stimulate cell differentiation from monocytes to Mφs. (**E** and **F**) Images (**E**) and quantification (**F**) of THP1 Mφ efferocytosis of apoptotic neutrophils (red, CellTracker) at 0, 1, 2, and 3 h. Scale bar: 20 μm. (**G**) Schematic of neutrophil efferocytosis assay in bone fracture muscle tissue by pMφs. (**H** and **I**) Effects of PDX treatment on neutrophil efferocytosis activity (**H**) and macrophage abundance (**I**) in the bone fracture tissue collected from WT and *Gpr37*^−/−^ mice (100 ng, i.v., *n* = 6). (**J** and **K**) Effects of PDX treatment on F4/80^+^ macrophage survival ratio using flow cytometry analysis from WT and *Gpr37*^−/−^ mice (100 ng, i.v., *n* = 6). Data are presented as mean ± SEM. Statistics: 1-way ANOVA with Bonferroni’s post hoc test (**B** and **F**) and 2-way ANOVA with Bonferroni’s post hoc test (**I** and **K**). *P < 0.05, **P < 0.01, ****P < 0.0001.

Macrophage efferocytosis of apoptotic cells is essential for tissue repair and inflammation resolution (36, 37). To test whether PDX promotes this process, we exposed pMφs to apoptotic, fluorescently labeled neutrophils. PDX significantly increased efferocytosis at 2 and 3 h compared with vehicle (Figure 8, A–C). PDX similarly enhanced efferocytosis in THP1 macrophages (Figure 8, D–F), consistent with its role in promoting resolution. PDX-induced efferocytosis was blocked by Ca²^+^ chelation (BAPTA-AM) and inhibited by pertussis toxin, a Gβ/γ blocker, and PI3K/AKT inhibition, indicating dependence on GPR37–Gi/o–Ca²^+^–PI3K signaling (Supplemental Figure 10C). To assess clearance in vivo, we quantified apoptotic neutrophils at fracture sites. PDX enhanced efferocytosis in WT mice but not in *Gpr37*^−/−^ mice (Figure 8, G–I). PDX also modestly improved macrophage survival in WT mice and reduced that in *Gpr37*^−/−^ mice (Figure 8, J and K). Thus, PDX promotes efferocytosis and macrophage survival through GPR37-dependent Ca²^+^ and Gi/βγ/PI3K-AKT signaling in vitro and in vivo.

### PDX inhibits C-fiber reflex and DRG neuron Ca^2+^ responses under fPOP.

**Figure 9:**
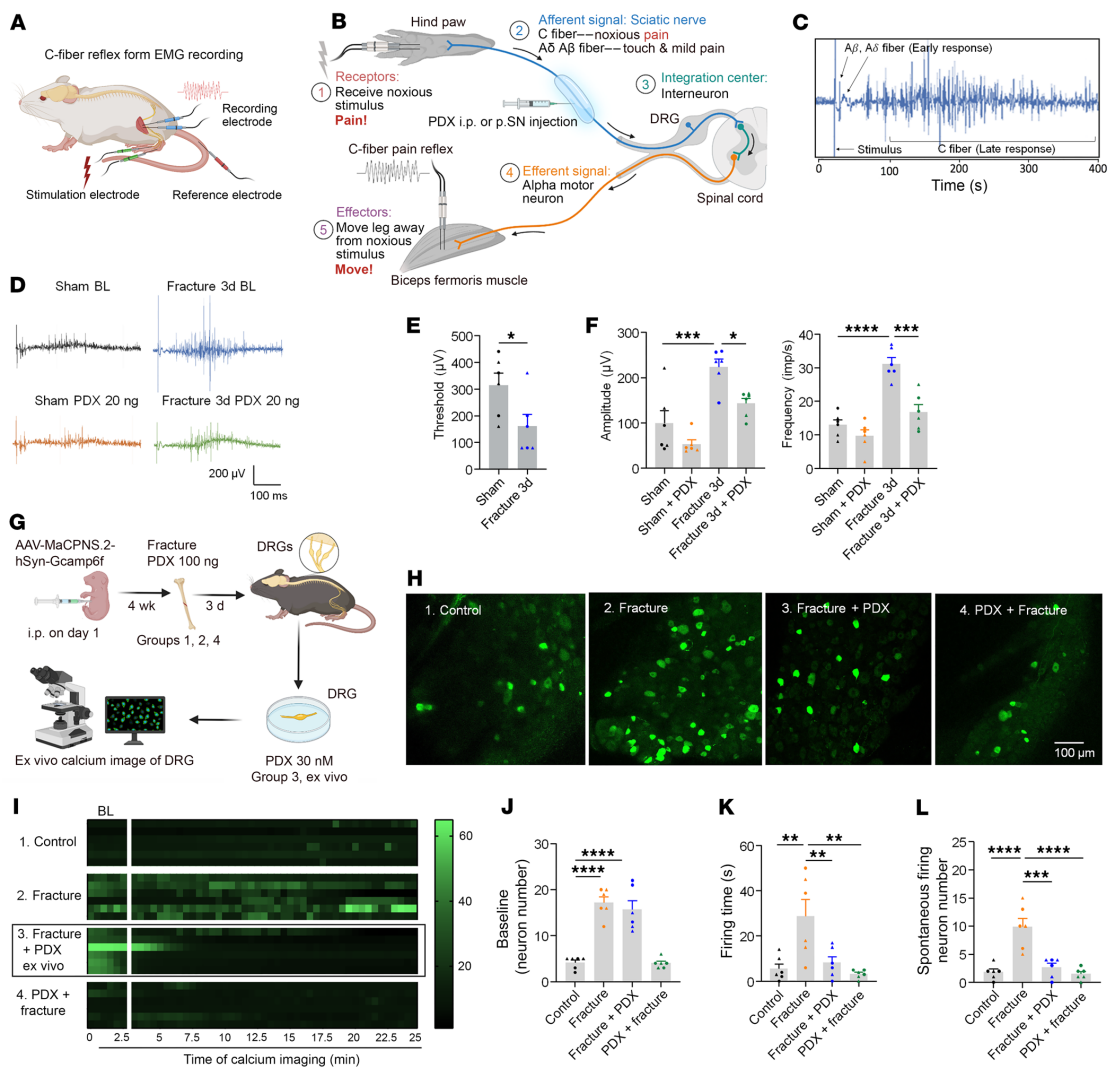
PDX inhibits the C-fiber reflex in vivo and DRG spontaneous neuronal activity ex vivo after fracture in CD1 mice. (**A** and **B**) Schematic illustrating EMG recording of the C-fiber reflex in the biceps femoris, including electrode placement (**A**) and the afferent–efferent spinal reflex arc (**B**). (**C**) Representative EMG traces showing A-fiber–mediated early responses and C-fiber–mediated late responses. (**D**) C-fiber reflex recordings in 4 groups: sham or fracture surgery with PBS or PDX. PDX (20 ng, 20 μL) was administered by p.SN. injection. EMG was recorded before (baseline) and 30 min after treatment. (**E**) Thresholds 3 days after sham and fracture surgery. (**F**) EMG amplitude (left) and frequency (right) from 4 groups in **D**. EMG data were quantified on postsurgical day 3. (**G**) Schematic of ex vivo whole-mount DRG calcium imaging in 4 treatment groups: sham (group 1), fracture (group 2), fracture with ex vivo PDX bath application (30 nM; group 3), and fracture with in vivo PDX pretreatment (100 ng i.v.; group 4). AAV-MaCPNS.2-hSyn-Gcamp6f was delivered at P1, and ipsilateral L3–L5 DRG was collected on day 3 after fracture for imaging using confocal microscopy. (**H** and **I**) Representative Ca^2+^ traces (**H**) and heatmaps (**I**) demonstrate that fracture induces spontaneous DRG neuronal activity, which is prevented by in vivo PDX and reversed by ex vivo PDX (*n* = 6 neuron/group). Scale bar: 100 μm. (**J**–**L**) Quantification of calcium signal in DRG neurons, showing number of neurons per DRG with spontaneous activity at the baseline (**J**), firing time of DRG neurons for all the firing events in each DRG (**K**), and the number of spontaneously discharging neurons in each DRG (**L**). Data are presented as mean ± SEM. Statistics: 1-way ANOVA with Bonferroni’s post hoc test. *P < 0.05, **P < 0.01, ***P < 0.001, ****P < 0.0001; *n* = 6 mice/group; triangles, male; circles, female.

RNA-seq of DRG from fracture mice showed that PDX downregulated muscle contraction and calcium-signaling pathways (Figure 6, A, I, and J), suggesting modulation of muscle nociception, an important component of postoperative pain. We assessed nociceptive C-fiber reflexes via biceps femoris electromyogram (Figure 9, A and B). Tibial fracture lowered reflex threshold and latency and increased amplitude and firing frequency (*P* = 0.0376; *P* < 0.001), consistent with sensitization (Figure 9, C–F). Perisciatic PDX (20 ng, 30 min) reversed these changes, and intraperitoneal PDX (150 ng) similarly reduced reflex amplitude and frequency (Supplemental Figure 11, A–D). Thus, PDX blocks fracture-induced C-fiber reflex facilitation.

To examine sensory neuron activity, we performed ex vivo Ca²^+^ imaging of L3/4 DRG expressing GCaMP6f (AAV-MaCPNS.2-hSyn-GCaMP6f; Figure 9G). Fracture increased baseline Ca²^+^ activity by day 3, including longer spontaneous discharge and more active neurons (Figure 9, H–L). Both pre- and posttreatment PDX markedly reduced spontaneous Ca²^+^ events and the number of hyperactive neurons (Figure 9, I–L). These results indicate that fracture induces DRG neuron hyperexcitability and enhances C-fiber reflexes, whereas PDX normalizes neuronal activity and suppresses nociceptive reflex facilitation.

### PDX inhibits TRPA1/TRPV1-induced pain and neurogenic inflammation.

**Figure 10:**
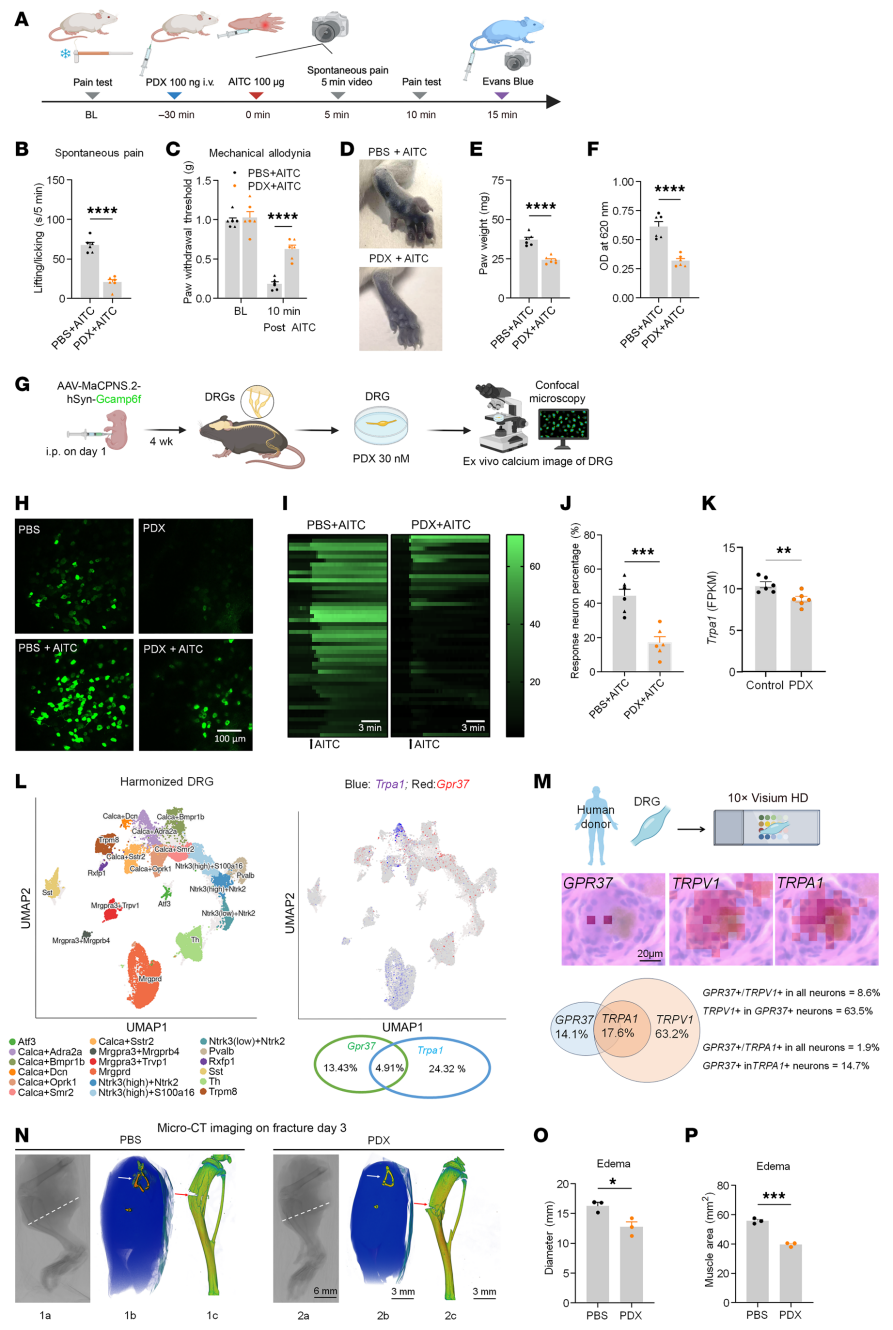
PDX reduces TRPA1-mediated spontaneous pain and neurogenic inflammation in CD1 mice. (**A**) Experimental scheme: mice received PDX 30 min before intraplantar AITC (100 μg); spontaneous pain was recorded, followed by i.v. Evans blue to assess neurogenic inflammation. (**B**) PDX reduced AITC-evoked spontaneous pain (5 min). (**C**) PDX decreased AITC-induced mechanical allodynia (von Frey, 10 min). (**D**) Representative hind paw images showing AITC-induced edema. (**E** and **F**) Quantification of edema by paw weight (**E**) and Evans blue extravasation (**F**) with and without PDX (100 ng, i.v.). (**G**) Schematic of ex vivo Ca^2+^ imaging in AAV-MaCPNS.2-hSyn-Gcamp6f–labeled neurons. (**H** and **I**) Representative Ca^2+^ responses (**H**) and heatmaps (**I**) showing AITC-evoked neuronal activation. Each group includes 51 DRG neurons. Scale bar: 100 μm. (**J**) PDX decreased the proportion of AITC-responsive DRG neurons. (**K**) Bulk RNA-seq revealed reduced *Trpa1* expression in DRG after fracture in PDX-treated mice. (**L**) Left, subcluster annotation map of mouse DRG. Right, UMAP plot showing *Gpr37* (red) and *Trpa1* (blue) expression. Published scRNA-seq data of mouse DRG (60) shows the percentages of *Gpr37*- and *Trpa1*-expressing neurons and their colocalization. (**M**) Spatial transcriptomics of human DRG showing colocalization of GPR37 with TRPA1 and TRPV1 in human sensory neurons. TRPA1 expression was confined to TRPV1^+^ neurons (1,062 neurons from 2 donors). Scale bar: 20 μm. (**N**) Micro-CT of hind limbs 3 days after fracture showed that PDX reduced edema and improved bone integrity. Coronal and 3D reconstructions revealed reduced soft-tissue swelling and enhanced tibial bone density at the fracture site. The rightmost PBS and PDX images show 3D constructed tibial bones, and the white and red arrows indicate the fracture sites. Scale bars: 6 and 3 mm. (**O** and **P**) Quantification of diameters (**O**), indicated by white lines in the leftmost PBS and PDX images in **N**, and coronal section muscle area (**P**), indicated in the middle PBS and PDX images in **N**. Data are presented as mean ± SEM. Statistics: unpaired *t* test (**B**, **E**, **F**, **J**, **K**, **O**, and **P**) and 1-way ANOVA with Bonferroni’s post hoc test (**C**). *P < 0.05, **P < 0.01, ***P < 0.001, ****P < 0.0001; *n* = 6 mice/group for **B**, **C**, **E**, **F**, **J**, and **K**; *n* = 3 males for **O** and **P**; triangles, male; circles, female.

Transient receptor potential ion channels A1 and V1 (TRPA1 and TRPV1), expressed by nociceptors, play a crucial role in nociception; furthermore, SPMs can modulate their activity (16, 38). To test whether PDX reduces TRPA1/V1 channel-evoked pain, we conducted intraplantar injection of TRPA1 agonist allyl isothiocyanate (AITC) (100 μg, intraplantar [i.pl.]) or TRPV1 agonist capsaicin (1 μg). PDX markedly suppressed AITC-evoked spontaneous and mechanical pain (*P* < 0.0001; Figure 10, A–C) and similarly reduced capsaicin-evoked pain behaviors (Supplemental Figure 12, A–C). PDX also diminished AITC- and capsaicin-induced paw edema, as indicated by Evans blue extravasation (*P* < 0.0001; Figure 10, D–F, and Supplemental Figure 12, D–F).

In DRG neurons, PDX decreased the proportion of AITC-responsive cells from 44.3% to 16.97% and capsaicin-responsive cells from 20.40% to 5.95% (Figure 10, G–J, and Supplemental Figure 12, G–I). RNA-seq in fracture DRG showed reduced *Trpa1* expression after PDX treatment (*P* < 0.01; Figure 10K). Single-cell and spatial transcriptomics confirmed coexpression of GPR37 with TRPA1 and TRPV1 in mouse and human DRG neurons, with TRPA1 exclusively in TRPV1^+^ cells (Figure 10, L and M, and Supplemental Figure 12, J and K). PDX did not directly alter TRPA1 currents in TRPA1-transfected HEK cells, suggesting indirect neuronal modulation (Supplemental Figure 13, A and B).

Finally, micro-CT analysis showed that PDX pretreatment significantly reduced fracture-induced hind-limb edema, as indicated by decreased limb diameter and muscle area (*P* < 0.001; Figure 10, N–P), and also lessened bone damage (Figure 10O). These findings suggest that PDX confers additional benefit by suppressing TRPA1/TRPV1-mediated neurogenic inflammation and protecting bone integrity.

## Discussion

**Figure 11:**
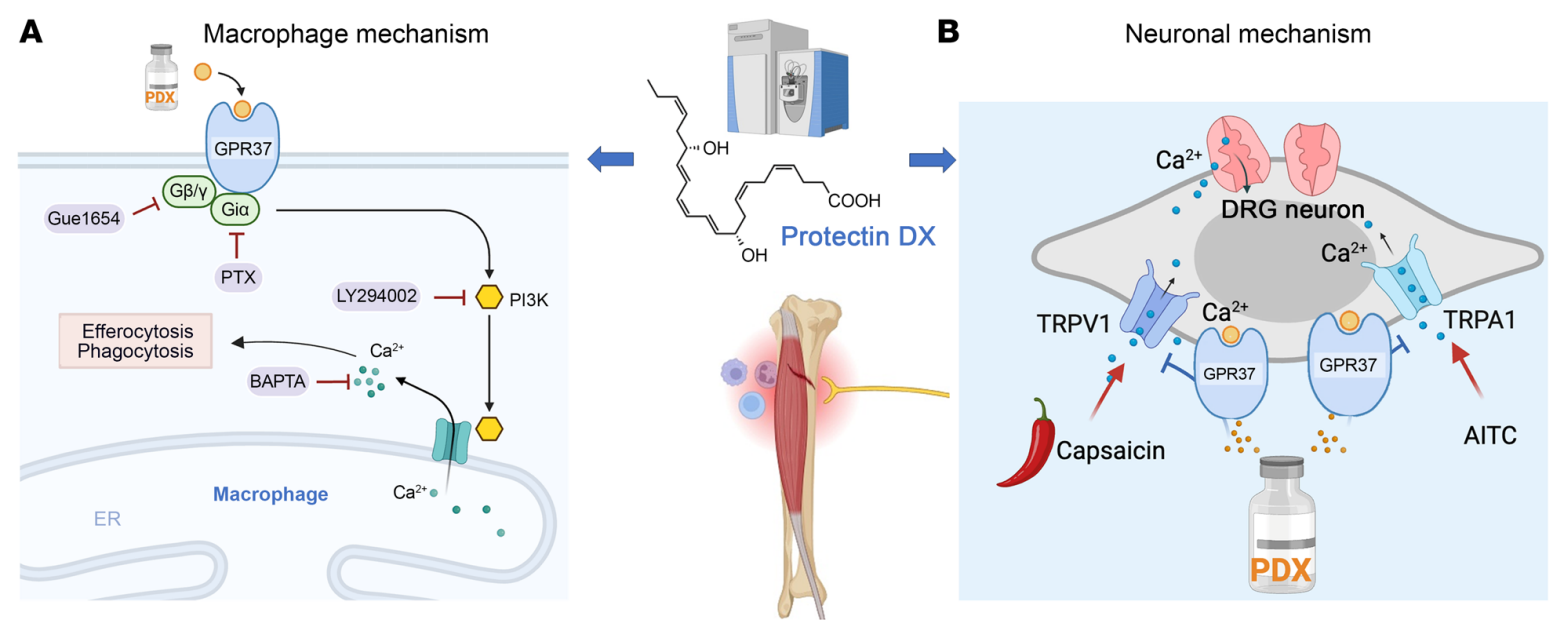
Working hypothesis by which PDX alleviates postoperative pain after tibial bone fracture via macrophage and neuronal mechanisms. (**A**) Schematic illustration of PDX-induced phagocytosis and efferocytosis in pMφs via Giα, Gβ/γ, and PI3K/AKT signaling pathways and intracellular calcium signaling. Macrophage mechanism by which PDX induces phagocytosis/efferocytosis and differentially regulates the expression of pro-inflammatory and anti-inflammatory cytokines. (**B**) Schematic of neuronal mechanism by which PDX inhibits TRPA1/TRPV1 and calcium signaling in nociceptor neurons via GPR37.

Earlier results demonstrated the anti-inflammatory actions of PDX, which promote macrophage polarization through PPARγ-dependent signaling pathways (39). PDX mitigates type 2 diabetes via activation of a myokine-liver glucoregulatory axis (25). In the present study, we demonstrated that PDX signals through GPR37 and enhances macrophage phagocytosis/efferocytosis using binding, in vitro, and in vivo assays, as well as *Gpr37*-KO animals. We have shown the crucial role of GPR37 in mediating the analgesic actions and the proresolution functions of PDX via macrophage and neuronal mechanisms (Figure 11, A and B).

Our findings provide mechanistic insights into macrophage signaling in pain. Mechanistically, PDX’s effect on macrophage phagocytosis and efferocytosis is highly dependent on intracellular calcium and requires the involvement of Giα, Gβ/γ, and the PI3K/AKT signaling pathway (Supplemental Figure 9, A and B). Efferocytosis plays a crucial role in tissue homeostasis to ensure the removal of apoptotic cells (e.g., neutrophils) in tissues, preventing the accumulation of dead cells. SPMs have been shown to promote inflammation resolution by enhancing phagocytosis and efferocytosis (40, 41). Our findings show that PDX triggers GPR37-mediated phagocytosis in peritoneal macrophages via calcium signaling, where the calcium chelator BAPTA-AM inhibits PDX-induced phagocytosis. Additionally, Gi signaling via GPR37 is necessary for phagocytosis, which completes within 60 min after initiation, while efferocytosis of apoptotic neutrophils, linked to inflammation resolution, occurs hours later. Our bulk RNA-seq results also demonstrate that PDX upregulates the S100 signaling pathway, enhancing genes associated with NO and ROS production in DRG macrophages, crucial for pathogen defense and macrophage migration, and influencing antiviral and antibacterial responses (Figure 6, A–F). Consistent with our findings, increased levels of the neutrophil markers S100a9 and S100a8 have been linked to the resolution of inflammatory pain in mice, while neutrophil activation is also associated with recovery from low-back pain in patients (6).

Growing evidence underscores the importance of macrophages in pain regulation. Macrophages promote pain through mediators like cytokines that interact with nociceptors (42–45), and their polarization under different conditions can both initiate and alleviate pain (46). Macrophages also facilitate the resolution of inflammatory pain and neuropathic pain through cytokine production (e.g., IL-10), phagocytosis, exosome secretion, or mitochondrial transfer (18, 47–49). While microglia predominantly regulate neuropathic pain in males (50), macrophages affect pain in both sexes, with studies revealing microglia-independent, macrophage-dependent peripheral sciatic pain in both male and female mice (51). Furthermore, the TLR9-mediated macrophage pathway regulates neuropathic pain in males (42), while the IL-23/IL-17–mediated macrophage signaling pathway modulates mechanical pain in females (52). Our research indicates that PDX effectively reduces postoperative pain in both sexes following both early and late treatments (Supplemental Figure 5, D and E). This is also reflected in the equal inclusion of male and female animals in most experiments. Sex and strain differences play an important role in pain processing (53, 54). We observed that female mice lacking GPR37 exhibit more severe and persistent cold pain compared with males, suggesting a chronic pain state driven by GPR37 deficiency in females. Additionally, we identified strain-specific differences in the resolution of postoperative pain: the duration of fPOP is shorter in CD1 mice than in C57BL/6 mice (2–3 weeks vs. 5–6 weeks). Heritability of nociceptive traits has been reported among inbred mouse strains, including C57BL/6J, across 12 nociceptive measures (54). CD1/ICR mice are outbred and generally exhibit more robust immune responses than inbred strains such as C57BL/6. Compared with C57BL/6 mice, immune cells from CD1 mice show heightened responsiveness to inflammatory stimuli (55, 56), which may contribute to accelerated fracture healing and shorter pain duration. CD1 mice also have larger body sizes and, in our hands, demonstrated greater resistance to LPS-induced septic death compared with C57BL/6 mice (57).

Furthermore, our studies have uncovered neuronal signaling by PDX in alleviating acute pain and neurogenic inflammation (Figure 11B). It is established that SPMs modulate TRPA1 and TRPV1 activities in mouse DRG sensory neurons (9, 16). Our results showed that PDX did not inhibit AITC-induced calcium responses in TRPA1-expressing cells (Supplemental Figure 8, D and E), indicating that PDX is not a direct TRPA1 antagonist. Instead, PDX may modulate TRPA1 and TRPV1 activity indirectly via GPR37 signaling in DRG neurons (Figure 11B), consistent with our previous reports showing that SPMs inhibit TRPA1 and TRPV1 through GPCR pathways (38, 58). Our RNA-seq data revealed that PDX treatment significantly reduced Trpa1 expression in the mouse DRG following bone fracture (Figure 10K), in support of our calcium imaging studies showing that PDX can significantly suppress bone fracture–induced calcium responses in DRG neurons ex vivo. To demonstrate translational relevance, our spatial transcriptomic analysis revealed colocalization of GPR37 with TRPA1 and TRPV1 in human DRG neurons, providing a cellular basis for GPR37-mediated regulation of these ion channels. By inhibiting both TRPA1 and TRPV1 pathways, PDX effectively reduced spontaneous pain, neurogenic inflammation, and calcium influx induced by AITC and capsaicin. Supporting this, pathway analysis and ex vivo calcium imaging demonstrated significant suppression of calcium signaling and reduced neuronal hyperactivity in DRG neurons following PDX treatment in the fPOP model (Figure 9J). Furthermore, in vivo electromyography confirmed that PDX attenuates C-fiber–evoked nociceptive muscle reflex responses. Future studies are warranted to investigate how GPR37 in sensory neurons regulates TRPA1 and TRPV1 signaling.

Notably, this research provides insights into the resolution physiology mediated by SPMs (59) (Figure 5G). Our lipidomic analysis revealed that PDX is one of the most abundantly produced SPM in muscle and spleen tissues following orthopedic surgery (Figure 3, A–F). Strikingly, PDX levels in muscle were approximately 10-fold higher than those of PD1, underscoring its potential importance in inflammation and pain resolution. We previously demonstrated potent analgesic actions of RvD1 and RvE1 in animal models of inflammatory pain (9); accordingly, we proposed the concept for the resolution of inflammation and pain by SPMs (8). Increasing evidence suggests anti-inflammatory treatments such as steroids and NSAIDs may delay the resolution of inflammatory pain (6, 8). Importantly, we found that treatment with dexamethasone and meloxicam delayed the resolution of fPOP, thereby extending pain recovery periods (Figure 5G). In contrast, PDX facilitated the resolution of fPOP by reducing pain intensity and shortening its duration. PDX concentrations are elevated in tissues by the influx of blood cells that carry PDX biosynthesis enzymes (22, 23). Additionally, our findings highlighted the pivotal role of GPR37 in resolving postoperative pain; in *Gpr37*^−/−^ mice, fPOP recovery was not observed within 3 months, indicating the development of chronic pain (60) (Figure 5G). Hence, our results support our initial hypothesis that unresolved acute pain can evolve into chronic pain (8). Our RNA-seq experiments, pathway analysis, and micro-CT imaging indicate that PDX regulates macrophage/neutrophil signaling, tissue regeneration, and wound healing. Intriguingly, recent research revealed a critical role for neutrophil activation in resolving inflammatory pain (6). Consistently, we found marked immune activation in the DRG of PDX-treated mice in the fPOP model (Figure 6A), as evidenced by the upregulation of the S100A family pathway, including *S100a8* and *S100a9* (Figure 6, A–G). Correspondingly, PDX treatment increased IL-10 expression and secretion from pMφs following LPS stimulation. As a key anti-inflammatory cytokine, IL-10 is critical for the resolution of pain (18).

Globally, over 310 million surgeries are performed each year, including 40 million orthopedic surgeries (61). Postoperative pain significantly affects recovery and quality of life, yet many patients report inadequate analgesia (62). Despite extensive research and advances in analgesic therapies, postoperative pain management remains insufficient (3, 31). A systematic review of major gastrointestinal surgeries found that preoperative use of omega-3 fatty acids may reduce hospital stay durations (63). However, these studies did not monitor the in vivo production of SPMs. In a large-scale ancillary study, middle-aged and older US adults that received moderate daily doses of omega-3 fatty acids did not experience reduction in the prevalence or severity of pain (64). A meta-analysis suggested that a higher daily dose of 2.7 g of omega-3s might be necessary to alleviate pain (65), yet these studies did not monitor in vivo SPM production. It is important to note that besides SPM production, these fatty acids undergo various metabolic processes in humans (66). Despite the limited efficacy of omega-3 fatty acids in pain management, our study demonstrates the superior analgesic potential of PDX via various administration routes (Supplemental Figure 7). Notably, mitigation of postoperative pain with extremely low intravenous doses of PDX (0.003 mg/kg, ~30 nM concentration) should not impact platelet function or increase bleeding risk. Furthermore, FDA-approved treatments such as the biased opioid agonist Olinvyk (TRV130, 0.1–0.5 mg/kg, i.v.) (67, 68) and VX-548 (60 mg, oral [p.o.]), a selective Na_V_1.8 sodium channel inhibitor, require much higher doses to alleviate postoperative pain (69).

This study has several limitations. While we identified GPR37 as a potential receptor for PDX, the involvement of other GPCRs in PDX-induced macrophage signaling cannot be ruled out. In sensory neurons, various GPCRs or ion channels could mediate PDX’s analgesic effects. Further research into the downstream signaling pathways of PDX in immune cells and neurons is warranted. A recent study demonstrated that PD1 restores myogenesis, enhances muscle regeneration following injury, and improves muscle phenotype in a dystrophic mouse model (70). These findings raise the intriguing possibility that PDX may similarly promote muscle regeneration through GPR37 signaling.

In conclusion, we highlighted a resolution physiology (59) and resolution pharmacology (16) in this study. PDX is endogenously produced and exerts potent analgesic and proresolution effects through GPR37 activation and coordinated macrophage and neuronal signaling (Figure 11, A and B). Notably, while anti-inflammatory treatments such as steroids and NSAIDs impair the resolution of fPOP, PDX promotes its resolution (Figure 5G). Given its enhanced potency, simpler production compared with PD1, and growing mechanistic understanding, PDX holds promise as a more effective and safer nonopioid therapeutic option for postoperative pain.

## Methods

### Sex as a biological variable.

This study used both male and female animals. The numbers of male and female animals used in the different experiments are presented in Supplemental Table 1.

### Animals.

Adult mice (8–16 weeks) and young animals (4–5 weeks, for DRG calcium imaging only) were used in this study unless stated otherwise. *Gpr37*^tm1Dgen^ (JAX 005806) mice were purchased from The Jackson Laboratory. CD1 mice (Charles River Laboratories) were used for behavioral, electrophysiological, and biochemical tests. C57BL/6 mice were only used in experiments involving knockout mice. Animals were randomly assigned to each group. All animals were maintained at the Duke University Animal Facility. Sample sizes were based on our previous studies with similar assays (18).

### Lipidomic identification of PD1 and PDX.

PD1 and PDX, used for in vivo and in vitro tests in this study, were purchased from Cayman Chemical and authenticated using UV spectroscopy and LC-MS/MS, by comparing the obtained UV spectra, retention times, and fragmentation patterns of the compounds with those of known standards.

To examine the production of SPMs in the muscle surrounding the tibia and the spleen following tibial bone fracture, muscle and spleen samples were collected 3 days after tibial bone fracture injury. Approximately 3 mg of muscle tissue from the fracture area and the entire spleen were immediately isolated and snap-frozen in liquid nitrogen. The tissue then underwent solid phase extraction, and resulting methanol fractions were analyzed using LC-MS/MS by matching retention times and fragmentation patterns to those of known standards. Details regarding LC-MS/MS equipment and conditions, mass spectra, and the UV spectra of PD1 and PDX are presented in Supplemental Figures 2 and 3 and Supplemental Tables 4 and 5.

### Mouse models of postoperative pain and acute inflammatory pain.

Tibial fracture was performed under isoflurane anesthesia, as described previously (20). Muscles were dissociated following an incision on the left hind paw. A 0.38 mm stainless steel pin was inserted into the tibia intramedullary canal, followed by the osteotomy. The incision was sutured with a nonabsorbable 6-0 silk suture. Acute inflammatory pain was induced by a single intraplantar injection of AITC (100 mg) or capsaicin (1 mg).

### Drug injection.

All reagents were dissolved in sterile PBS and injected using a Hamilton microsyringe with a 30-gauge needle under brief isoflurane anesthesia. The drug volumes were 10 μL for local i.pl. and intrathecal (i.t.) injections, 20 μL for perisciatic nerve (p.SN.) injection, and 100 μL for i.v. and i.p. injection. For p.o., PDX (200 μL) was given by a reusable oral gavage needle (20G*50 mm).

### Behavioral tests for evoked pain and spontaneous pain in mice.

Mechanical pain was assessed using von Frey filaments. Animals were habituated to the testing environment daily for at least 2 days before baseline assessment. The room temperature and humidity remained stable for all experiments. To test mechanical sensitivity, we confined mice in boxes placed on an elevated metal mesh floor and stimulated their hind paws with a series of von Frey hairs with logarithmically increasing stiffness (0.02–2.56 g; Stoelting), presented perpendicularly to the central plantar surface. We determined the 50% PWT using Dixon’s up-and-down method. Thermal sensitivity was tested using a Hargreaves radiant heat apparatus (IITC Life Science). The basal paw withdrawal latency was adjusted to 9 to 15 seconds, with a cutoff of 20 seconds to prevent tissue damage. To test cold sensitivity, a drop of acetone was applied to the plantar surface of a hind paw, and the mouse’s response was observed for 60 seconds after acetone application. Paw flicking and licking time (seconds) to acetone was recorded. For fracture-induced spontaneous pain, we measured the time (seconds) mice spent licking or flinching the affected hind paws over 2 min after the fracture surgery at multiple time points. For capsaicin and AITC-induced spontaneous pain, we measured the time (seconds) mice spent licking or flinching the affected hind paws over 5 min after the capsaicin injection (1 μg, i.pl.) or AITC (100 μg, i.pl.). Behavioral assessments were conducted in a blinded manner.

### Dot blot assay for lipid-protein binding.

Lipid membrane coating and protein overlay assay were conducted as previously described (18). More details are provided in Supplemental Methods.

### ELISA.

ELISA for IL-1β, TNF-α, and IL-10 was conducted according to the manufacturer’s instructions. More details are provided in Supplemental Methods.

### RT-qPCR.

RT-qPCR analysis of *Il1b* and *Tnf* was conducted as previously described (18). More details are provided in Supplemental Methods.

### Bulk RNA-seq and pathway analysis of DRG tissues.

Total RNA was extracted from L3–L5 DRG and used to prepare mRNA libraries with the RNeasy Mini Kit (Qiagen, catalog 74104). All samples met quality criteria (RNA integrity number > 8.0, 260/280 > 2.0). Sequencing was performed on the Illumina NovaSeq 6000. Differential expression and pathway analysis were conducted to compare PDX- and vehicle-treated fracture groups. Pathway enrichment was assessed using gene ratio and Fisher’s exact test. Results were visualized with a volcano plot (Supplemental Table 2). Detailed protocols are provided in the Supplemental Methods.

### Phagocytosis and efferocytosis assay in pMϕs and THP1 cells.

The phagocytosis assay was modified from a previously described protocol (18). pHrodo Red zymosan bioparticles (Thermo Fisher Scientific, catalog P35364) were rinsed and reconstituted in RPMI medium. Zymosan particles (20 mg/mL) were added to cultures of pMφs or THP1 cells. After 0, 15, 30, 45, and 60 min of incubation with beads at 37°C, nonadherent beads were removed with cold PBS, and cells were fixed with 4% paraformaldehyde for 10 min. For neutrophil efferocytosis analysis, we collected whole blood (5 mL from 5 mice) to extract neutrophils. Mouse neutrophils were marked by CellTracker Red CMTPX Dye (Thermo Fisher Scientific, catalog C34552) and followed by UV light exposure for 10 min (250 mW/cm²) to induce neutrophil apoptosis. We then added the apoptotic neutrophils (5,000 cells) to the cultured pMφs or THP1 cells. At 0, 1, 2, and 3 h after the incubation at 37°C, nonadherent neutrophils were removed with cold PBS, and the remaining cells were fixed with 4% paraformaldehyde for 10 min. Three optical fields were photographed using a Zeiss 780 confocal microscope for quantification of zymosan particles or neutrophils ingested by Mφs, conducted on 50 to 400 pMφs or THP1 cells per condition or well, and triplicates were included for statistical analysis. FITC-conjugated antimouse F4/80 antibody (1:400, Sigma-Aldrich, MAPF152D) was used to mark pMφs, and FITC-conjugated antihuman CD45 antibody (1:400, BioLegend, 304054) was used to mark THP1 pMφs.

### Flow cytometry analysis of efferocytosis in bone fracture tissue.

WT and *Gpr37-*KO mice were subjected to bone fracture, and muscle tissue surrounding the fracture site was harvested 3 days after injury. Data were analyzed using Cytobank software (https://www.cytobank.org/cytobank). The gating strategy is illustrated in Supplemental Figure 14. More details are provided in Supplemental Methods.

### Spatial transcriptomic analysis in human DRG.

Human DRGs were obtained from donors through the National Disease Research Interchange with exemption permission from the Duke University IRB. Spatial transcriptomic analysis was conducted using Visium HD Spatial Gene Expression Reagent Kits (CG000686). More details are provided in Supplemental Methods.

### Micro-CT.

Micro-CT analyses were performed on the tibial bone from fracture mice using a Nikon XTH 225 ST scanner with the 225 kV source (Scanco), as previously described (71). Micro-CT data were quantified to assess the fractured leg’s diameter and cross-sectional area as an indication of edema on day 3 after fracture.

### Statistics.

All data in the figures are expressed as the mean ± SEM. The sample size for each experiment was based on our previous studies involving such experiments (18). Statistical analyses were performed using GraphPad Prism 10.2 (GraphPad Software). Biochemical and behavioral data were analyzed using a 2-tailed Student’s *t* test (unpaired, 2-group comparisons), 1-way ANOVA followed by Bonferroni’s or Tukey’s post hoc test, or 2-way ANOVA followed by Bonferroni’s or Tukey’s post hoc test. For the time-course analyses, repeated measures of ANOVA were used, when applicable. The criterion for statistical significance was a *P* value of less than 0.05. More details of statistical results are included in Supplemental Table 3, and the raw data are included in the Supporting Data Values file.

### Study approval.

The IACUC of Duke University approved all the animal procedures. Animal experiments were conducted in accordance with the NIH Guide for the Care and Use of Laboratory Animals.

### Data availability.

No custom software was used in this study. Values for all data points in graphs are reported in the Supporting Data Values file. RNA-seq data have been deposited in the Gene Expression Omnibus under accession number GSE309530.

## Author contributions

YL and RRJ developed the project. YL, SB, JX, ML, and SC conducted experiments and data analyses. RRJ, CNS, and YL participated in project development. JJ conducted the MS analysis of PD1, PDX, and SPMs under the guidance of CNS. YL and RRJ wrote the manuscript, and the other coauthors edited the manuscript.

## Funding support

This work is the result of NIH funding, in whole or in part, and is subject to the NIH Public Access Policy. Through acceptance of this federal funding, the NIH has been given a right to make the work publicly available in PubMed Central.

- Duke University Anesthesiology Research Funds (to RRJ).
- NIH grants R01NS131812, R61NS138215, and UG3NS143650 (to RRJ).
- Department of Defense grants W81XWH2110885, W81XWH2110756, W81XWH-2210267, and W81XWH-2210646 (to RRJ).
- NIH R03 grant DE032394 (to SB).
- NIH/National Institute of General Medical Sciences grant R35GM139430 (to CNS).

## Supplementary Material

Supplemental data

Unedited blot and gel images

Supplemental table 1

Supplemental table 2

Supplemental table 3

Supplemental video 1

Supplemental video 2

Supporting data values

## Version 1 01/16/2026

Electronic publication
