# Supplementary material for: Protectin DX resolves fracture-induced postoperative pain in mice via neuronal signaling and GPR37-activated macrophage efferocytosis
Source: J Clin Invest. 2026 Jan 16;136(2):e190754. doi: 10.1172/JCI190754 (PMC12807480; doi:10.1172/JCI190754)
Supplement: Supplemental data [file jci-136-190754-s139.pdf]

Protectin DX resolves fracture-induced postoperative pain in mice via neuronal signaling and GPR37-activated macrophage efferocytosis

Yize Li, Sangsu Bang, Jasmine Ji, Jing Xu, Min Lee, Sharat Chandra, Charles N Serhan, Ru-Rong Ji

Supplemental Figures (1-14)

Supplemental Tables (1-5)

Supplemental Materials and Methods

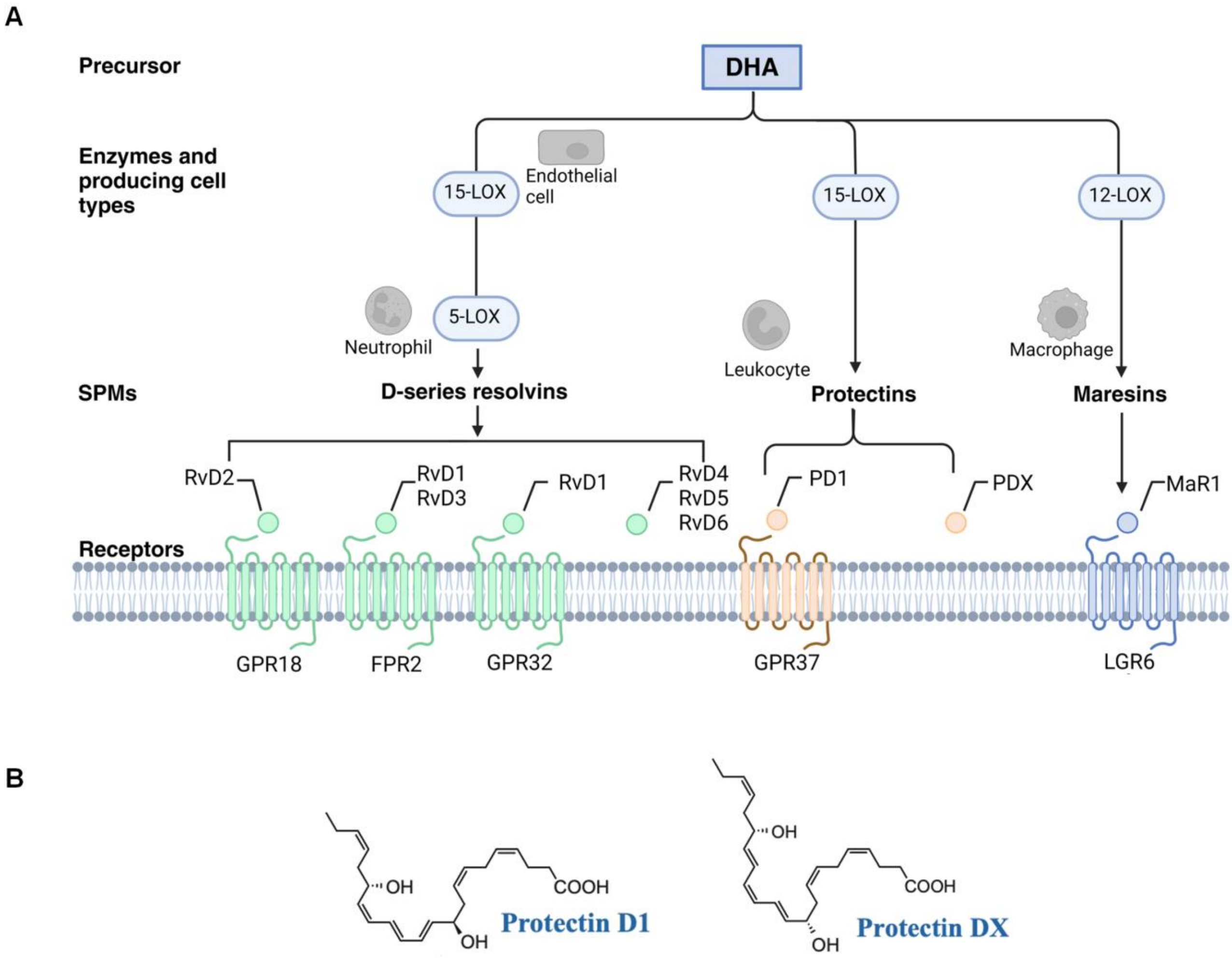

Supplemental Figure 1. Biosynthesis of SPMs, NPD1, and PDX from omega-3 polyunsaturated fatty acids.

(**A**) Schematic illustration of the pathway by which DHA (docosahexaenoic acid), a crucial precursor, is converted into specialized pro-resolving mediators (SPMs) through enzymatic reactions. The SPMs are classified into three main groups: D-series resolvins, protectins, and maresins. SPMs are produced by the combined actions of 15, 12, and 5 lipoxygenase (15-LOX, 12-LOX, 5-LOX) that interact with specific GPCRs, such as GPR18, FPR2, GPR32, GPR37, and LGR6, on immune cells, glial cells, and neurons. By binding to these receptors, SPMs help resolve inflammation and pain, facilitate tissue repair, and maintain homeostasis in the body (1-4). (**B**) Structures of NPD1/PD1 (10R,17S-dihydroxy-4Z,7Z,11E,13E,15Z,19Z-DHA) and PDX (10(S),17(S)-dihydroxy-4Z,7Z,11E,13Z,15E,19Z-DHA).

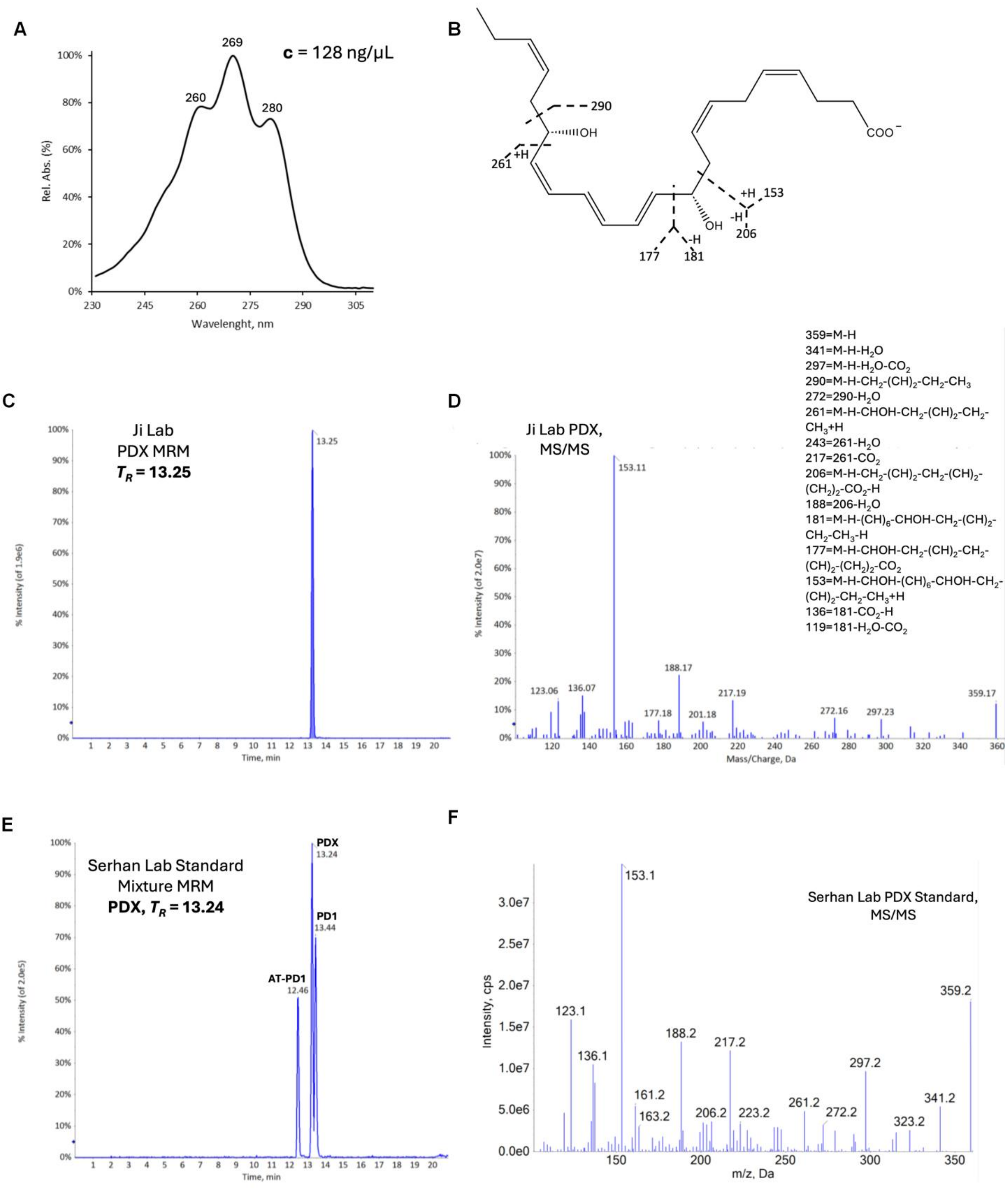

Supplemental Figure 2. Authentication of PDX from Ji Lab (purchased from Cayman Chemical) in Serhan Lab.

(**A**) UV absorbance spectrum of PDX at a concentration of 128 ng/μL (indicated as 100 ng/μL from Cayman). (**B**) structures of PD1. (**C-F**) Mass spectrometry (MS/MS) spectrum showing time (C, E) and mass/charge (D,

F) to represent the fragmentation pattern of PDX from Ji lab (C, D) and Serhan lab (E, F) using Serhan Lab Standard.

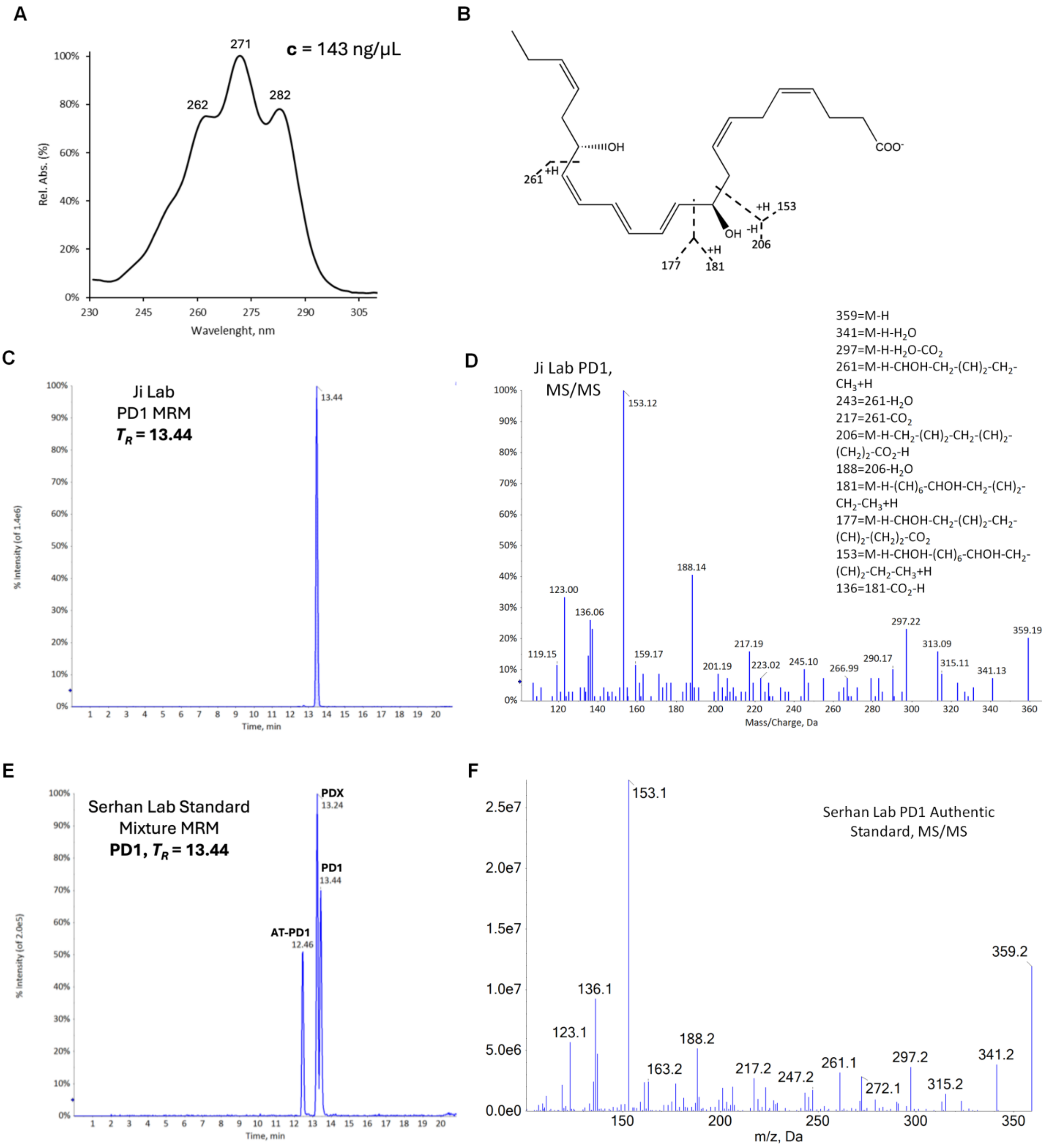

Supplemental Figure 3. Authentication of PD1 from Ji Lab (purchased from Cayman Chemical) in Serhan Lab.

(**A**) UV absorbance spectrum of PD1 at a concentration of 143 ng/μL (indicated as 100 ng/μL from Cayman). (**B**) structures of PD1. (**C-F**) Mass spectrometry (MS/MS) spectrum showing time (C, E) and mass/charge (D, F) to represent the fragmentation pattern of PD1 from Ji lab (C, D) and Serhan lab (E, F) using Serhan Lab Standard.

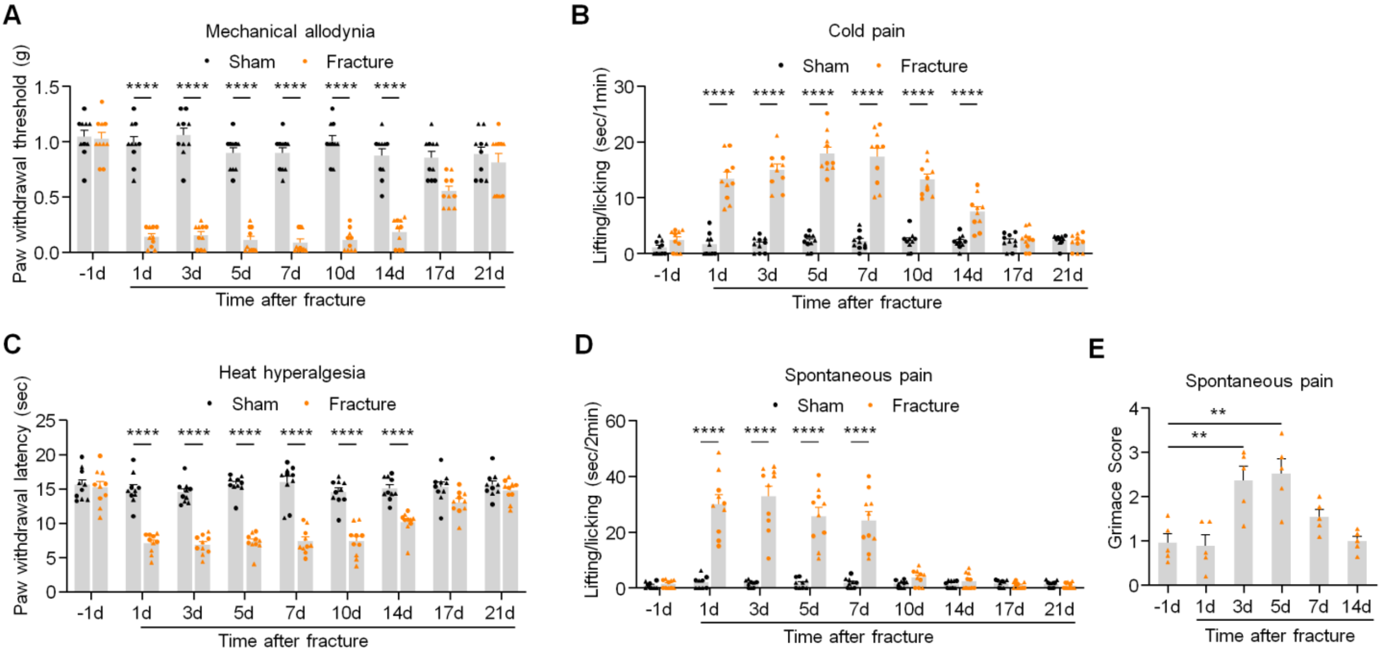

Supplemental Figure 4. Time course of sham surgery and tibial fracture induced postoperative pain in CD1 mice.

(**A**) Development and recovery of mechanical allodynia in von Frey test. (**B**) Development and recovery of cold allodynia in acetone test. (**C**) Development and recovery of heat hyperalgesia in Hargraves' test. (**D**) Development and recovery of spontaneous pain as time spent on lifting/licking behavior. (**E**) Tibial fracture induced spontaneous pain evaluated by Grimace score. Data are represented as mean ± SEM and statistically analyzed by two-way ANOVA or one-way ANOVA with Bonferroni's post hoc test. **$P < 0.01$, ****$P < 0.0001$; $n = 10$ mice (5 males and 5 females, A-D), n = 5 males (E). ▲ male, ● female.

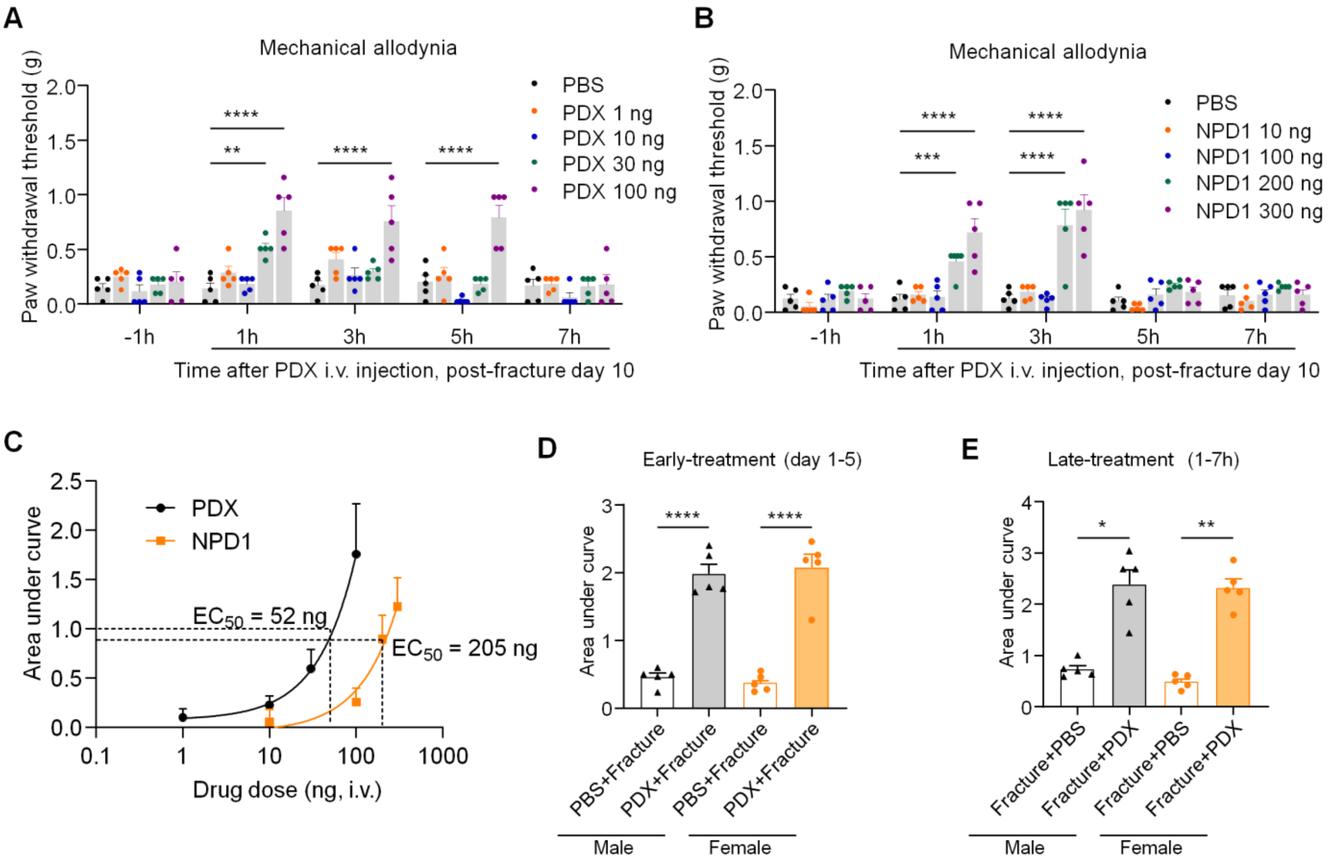

Supplemental Figure 5. Dose-dependent effects of PDX and NPD1 in fPOP in CD1 mice.

(**A**) Effects of i.v. injection of PDX (1, 10, 30, and 100 ng) and PBS (vehicle) on mechanical allodynia, revealed by PWT in von Frey tests on post-surgical day 10. n = 5 female mice per group. (**B**) Effects of i.v. injection of NPD1 (10, 10, 200, and 300 ng) and PBS on mechanical allodynia on post-surgical day 10. n = 5 female mice per group. (**C**) Comparison of AUC curves of PWT after PDX and NPD1 treatment, showing distinct EC_{50} values for PDX (52 ng) and NPD1 (205 ng). n = 5 female mice per group. (**D**) Comparison of AUC of PWT for PDX pre-treatment in males (black) and females (orange). Data were collected on post-surgical days 1, 3, and 5. n = 5 mice per sex per group. (**E**) Comparison of AUC of PDX post-treatment in males (black) and females (orange). Note that PDX is highly effective in reducing postoperative pain in both sexes. Data were collected from 1h, 3h, 5h, and 7h after PDX treatment on post-surgical day 10. n = 5 mice per sex per group. Data are represented as mean ± SEM and statistically analyzed by two-way ANOVA (A, B) and one-way ANOVA (C, D) with Bonferroni's post hoc test. $^*P < 0.05$, $^{**}P < 0.01$, $^{***}P < 0.001$, $^{****}P < 0.0001$; ▲ male, ● female.

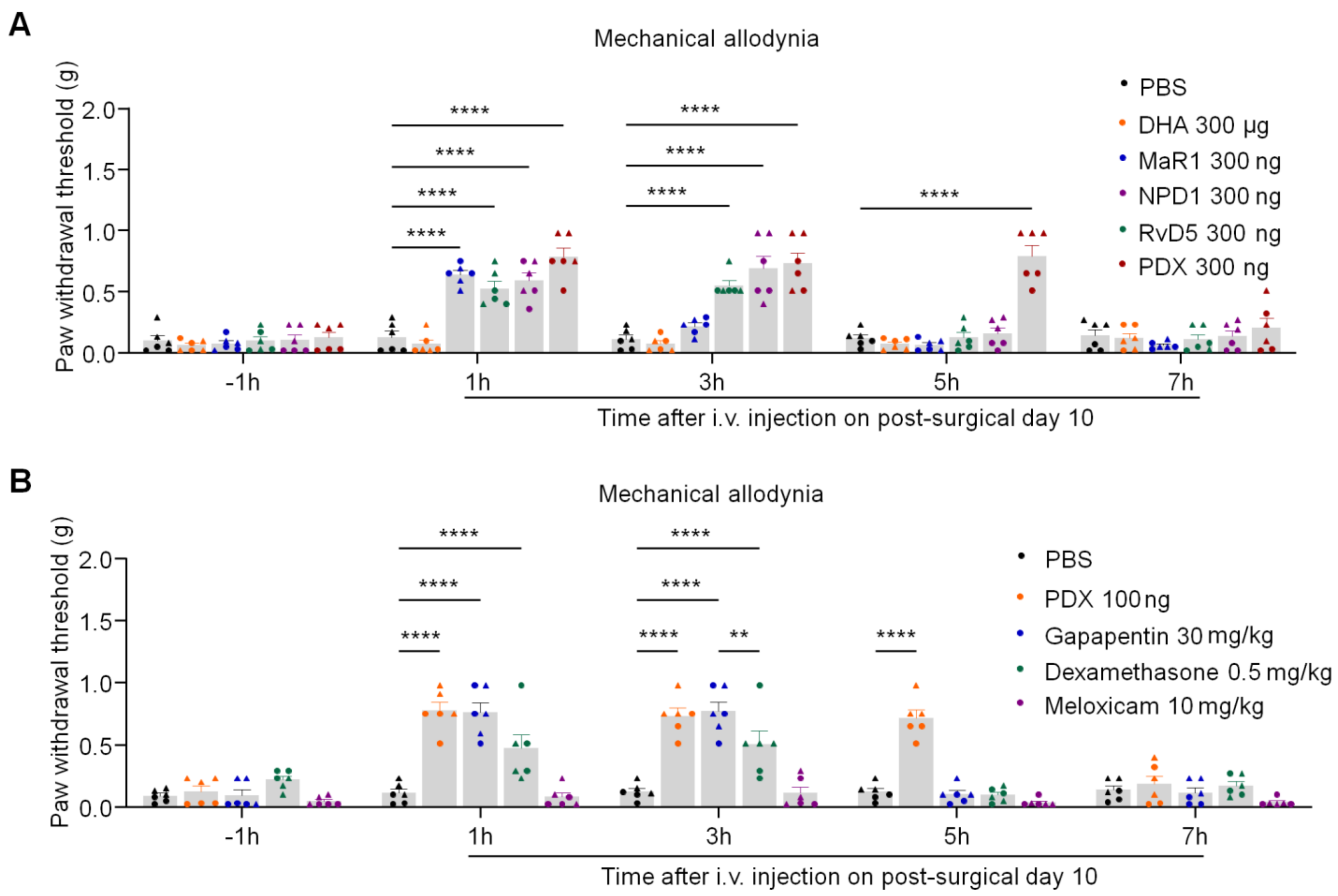

Supplemental Figure 6. Comparison of PDX-induced analgesia with MaR1, NPD1, RvD5, DHA, dexamethasone, and gabapentin in fPOP in CD1 mice.

(**A**) Effects of i.v. injection of DHA (300 μg), MaR1 (300 ng), NPD1 (300 ng), RvD5 (300 ng), and PDX (300 ng) on mechanical allodynia on fracture day 10. (**B**) Effects of i.v. injection of PDX (100 ng ~3 μg/kg), gabapentin (30 mg/kg), dexamethasone (0.5 mg/kg), and meloxicam (10 mg/kg) on mechanical allodynia on fracture day 10. Note that PDX is much more potent than gabapentin, dexamethasone, and meloxicam in reducing postoperative pain. Data are represented as mean ± SEM and statistically analyzed by two-way ANOVA with Bonferroni's post hoc test. ***$P < 0.01$, *****$P < 0.0001$; $n = 6$ mice per group (3 males and 3 females); ▲ male, ● female.

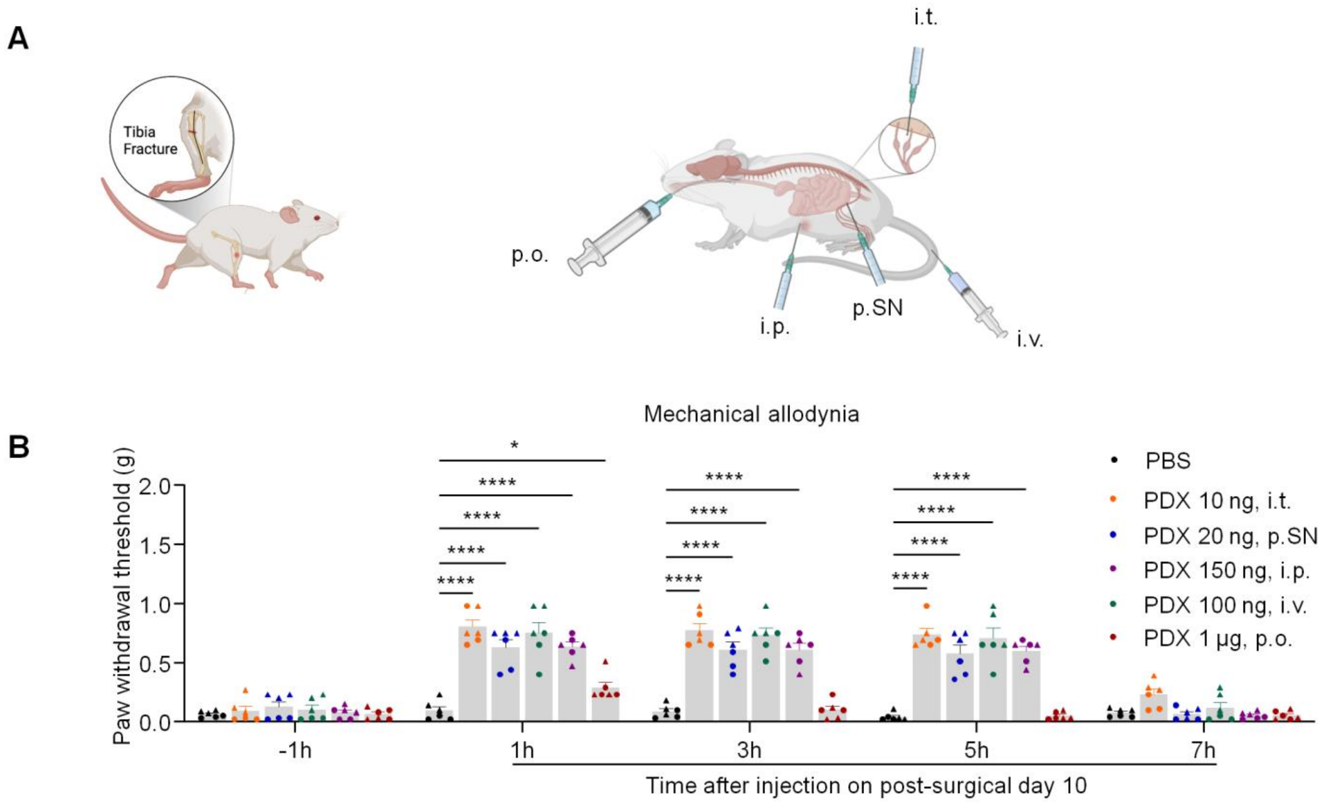

Supplemental Figure 7. Comparison of PDX-induced analgesia in fPOP via different administration routes in CD1 mice.

(A) Schematic of administration routes by which PDX inhibits postoperative pain. **(B)** Mechanical allodynia was assessed by PWT in von Frey tests, and PDX was administrated by intrathecal (i.t., 10 ng), peri-sciatic nerve (p.SN, 20 ng), intravenous (i.v., 100 ng), intraperitoneal (i.p., 150 ng), and oral gavage (p.o., 1 μg) routes on post-surgical day 10. Note that oral administration of PDX had only mild and transient pain relieving effects, even at a much higher dose. Data are represented as mean ± SEM and statistically analyzed by 2-way ANOVA with Bonferroni's post hoc test. *$P < 0.05$, ****$P < 0.0001$; $n = 6$ mice per group (3 males and 3 females); ▲ male, ● female.

A

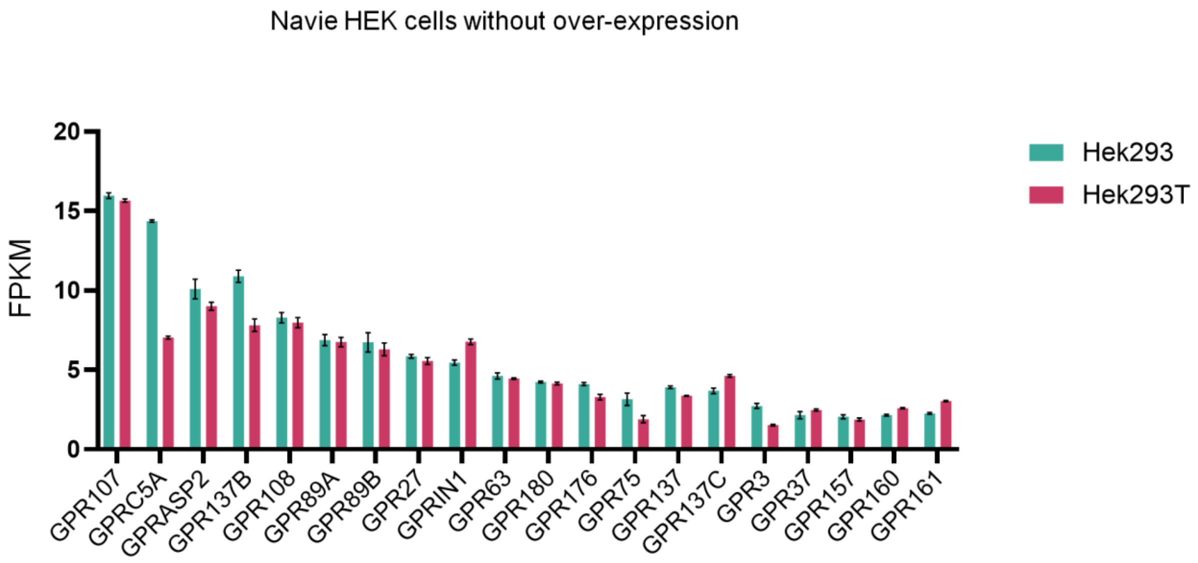

B

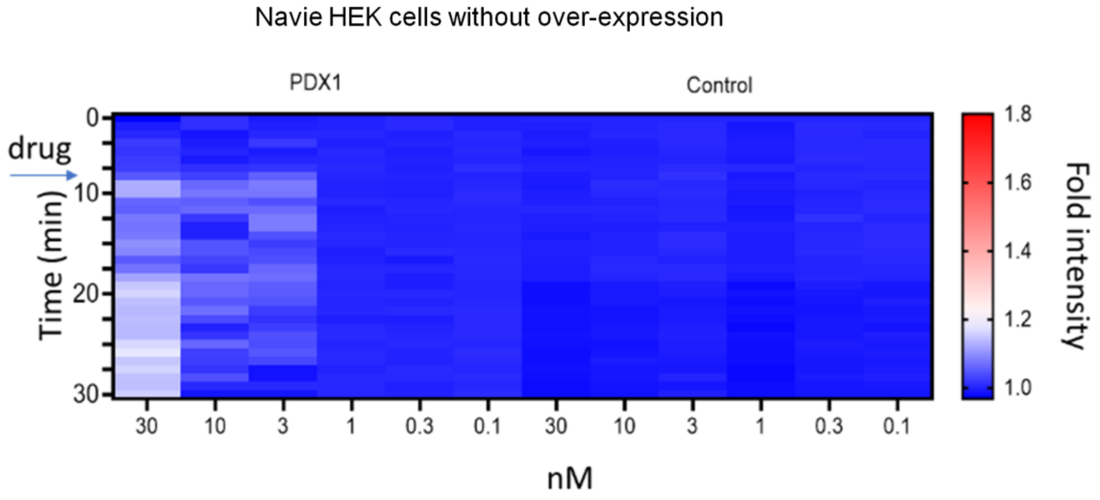

C

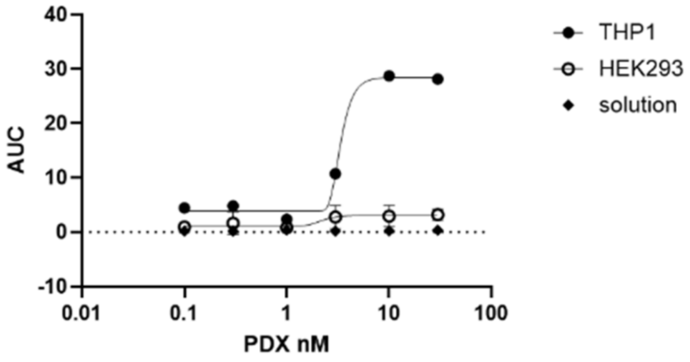

Supplemental Figure 8: **Characterization of basal constitutive activity of GPR37 expression in HEK293 cells.**

(A) Database analysis showing mRNA expression (FKPM value) of GPCRs in HEK293 and HEK293T cells (5). Notably, GPR37 expression is low compared to other GPCRs. (B) Native HEK cells exhibited no

detectable response to PDX at low concentrations. Even at 30 nM, only ~20% of cells showed a calcium response. (C) A dose–response experiment showing minimal Ca^{2+} signaling in naïve HEK cells. Notably, PDX induces robust Ca^{2+} response in THP1 cells, serving as a positive control (n =3).

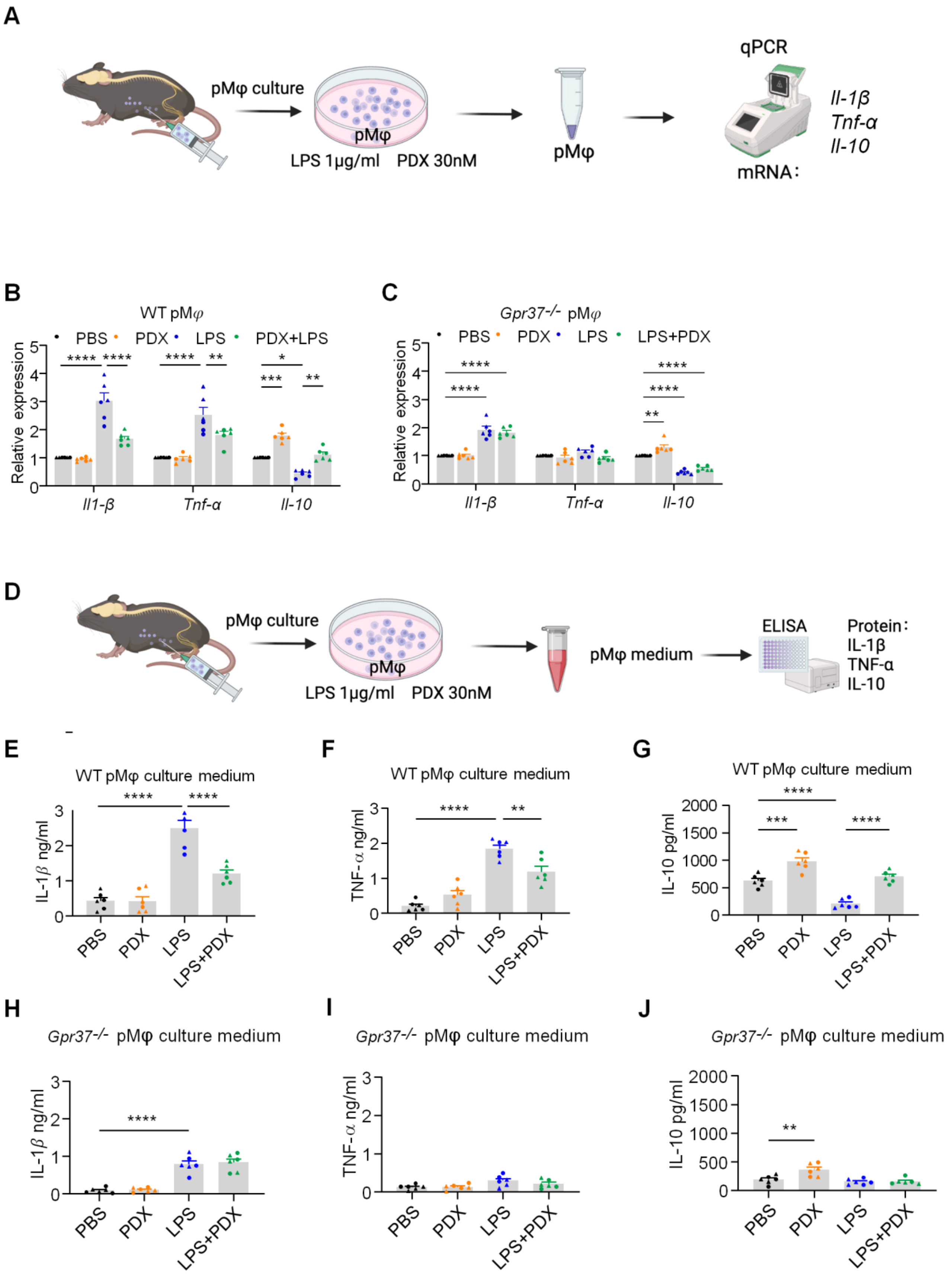

Supplemental Figure 9. PDX regulates the expression of pro- and anti-inflammatory cytokines in peritoneal macrophages of C57BL/6 mice via GPR37.

(A) Schematic of primary cultures for pMφ. Cultured cells were treated with LPS (1 μg/ml) and PDX (30 nM)

for 24 hours and collected for qRT-PCR analysis for *Il1b*, *Tnf,* and *Il10* expression. (**B, C**) Relative mRNA expression levels of *Il1b*, *Tnf,* and *Il10* after LPS or PDX treatment in pMφ cultures prepared from WT mice (B) and *Gpr37*$^{-/-}$ mice (C). **(D)** Schematic of primary cultures for pMφ. Cultured cells were treated with LPS (1 μg/ml) and PDX (30 nM) for 24 hours, and the collected media to tested for ELISA IL-1β, TNF-α, and IL-10 expression. (E-**G**) Media protein levels of IL-1β (E), TNF-α (F), and IL-10(J) after LPS or PDX treatment in pMφ cultures prepared from WT mice. (**H-J**) Media protein levels of IL-1β (H), TNF-α (I), and IL-10 (J) after LPS or PDX treatment in pMφ cultures prepared from *Gpr37*$^{-/-}$ mice. Data are represented as mean ± SEM and statistically analyzed by 2-way ANOVA with Bonferroni's post hoc test (B, C) and 1-way ANOVA with Tukey's post hoc test (E-J). *$P < 0.05$, **$P < 0.01$, ***$P < 0.001$, ****$P < 0.0001$; $n = 6$ cultures from 6 mice (3 males and 3 females); ▲ male, ● female.

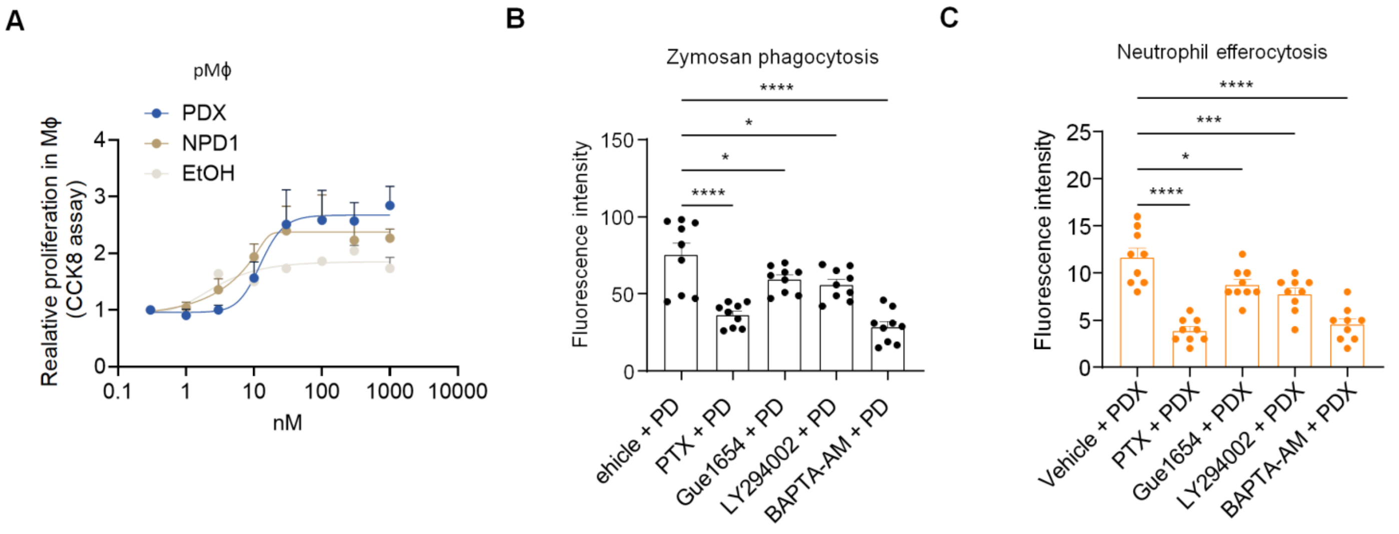

Supplemental Figure 10. Effects of PDX on proliferation, phagocytosis, and efferocytosis of peritoneal macrophages of CD1 mice.

(A) Dose-dependent effects of PDX and PD1 (0.3 nM to 1 μM) on macrophage proliferation activity in peritoneal macrophage culture using CCK8 assay. EtOH (0.3 nM to 1 μM) was used as a vehicle. Four cultures were prepared from 4 mice. EC_{50} = 12.5 nM for PDX. (**B, C**) Effects of PTX (1 μg/ml), Gue1654 (10 μM), LY294002 (20 μM), and BAPTA-AM (50 μM) on PDX (30 nM) induced zymosan phagocytosis (B) and neutrophil efferocytosis (C). $n = 9$ cultures/group. For each culture, the fluorescence intensity of zymosan and neutrophil uptake was analyzed by a plate reader. Data are represented as mean ± SEM and statistically analyzed by one-way ANOVA with Bonferroni's post hoc test. *$P < 0.05$, ***$P < 0.001$, ****$P < 0.0001$.

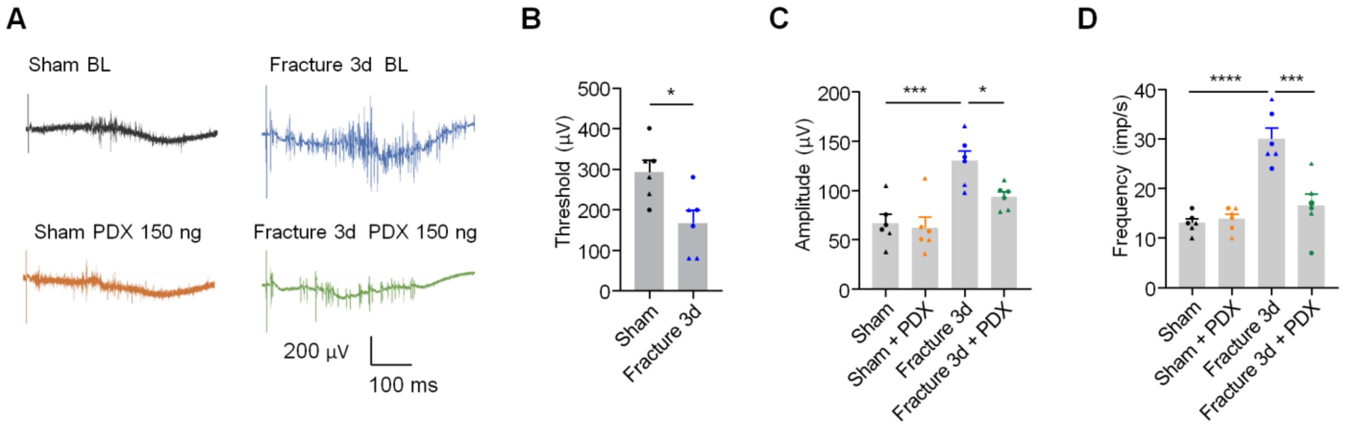

Supplemental Figure 11. Intraperitoneal injection of PDX decreases the C-fiber reflex fPOP.

(**A**) EMG traces of C-fiber reflex from 4 groups of animals with sham or fracture surgery with PBS and PDX. PDX (150 ng) was given by peritoneal injection. EMG recording was performed before (BL, baseline) and 30 min after the PDX treatment. (**B**) EMG threshold 3 days after sham and fracture surgery. (**C, D**) EMG amplitude (C) and frequency (D) from 4 groups in A. Data are represented as mean ± SEM and statistically analyzed by one-way ANOVA with Bonferroni's post hoc test. *$P < 0.05$, ***$P < 0.001$, ****$P < 0.0001$; n = 6 mice per group (3 males and 3 females); ▲ male, ● female.

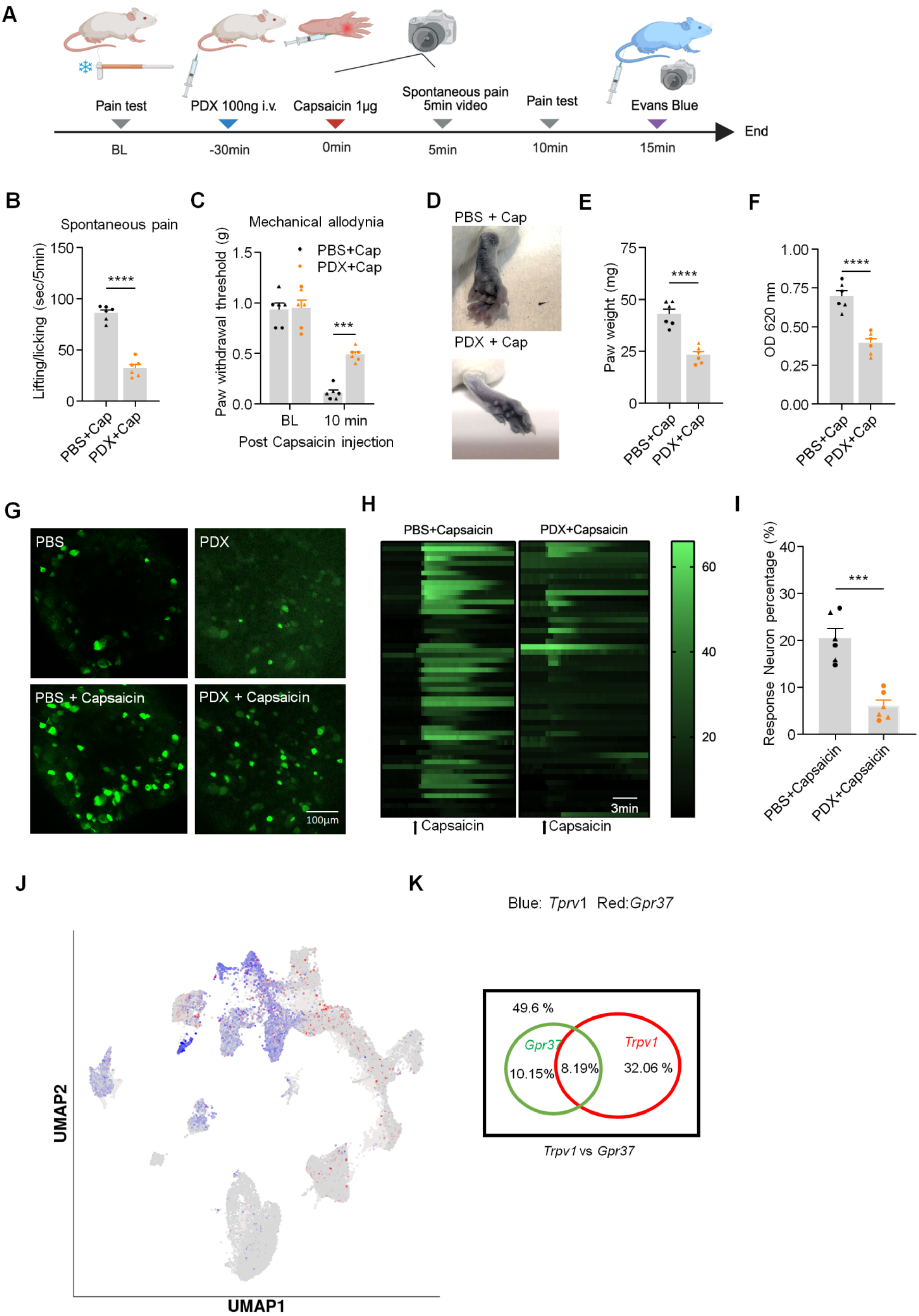

A
Pain test
PDX 100ng i.v.
Capsaicin 1μg
Spontaneous pain
5min video
Pain test
Evans Blue
End
BL
-30min
0min
5min
10min
15min
B
Spontaneous pain
Lifting/licking (sec/5min)
150
100
50
0
****
PBS+Cap
PDX+Cap
C
Mechanical allodynia
Paw withdrawal threshold (g)
1.5
1.0
0.5
0.0
PBS+Cap
PDX+Cap
***
BL
10 min
Post Capsaicin injection
D
PBS + Cap
PDX + Cap
E
Paw weight (mg)
75
50
25
0
****
PBS+Cap
PDX+Cap
F
OD 620 nm
1.00
0.75
0.50
0.25
0.00
****
PBS+Cap
PDX+Cap
G
PBS
PDX
PBS + Capsaicin
PDX + Capsaicin
100μm
H
PBS+Capsaicin
PDX+Capsaicin
60
40
20
3min
Capsaicin
Capsaicin
I
Response Neuron percentage (%)
40
30
20
10
0
***
PBS+Capsaicin
PDX+Capsaicin
J
UMAP2
UMAP1
K
Blue: Tprv1 Red:Gpr37
49.6 %
Gpr37
Trpv1
10.15%
8.19%
32.06 %
Trpv1 vs Gpr37

Supplemental Figure 12. PDX reduces TRPV1-mediated spontaneous pain and neurogenic inflammation in CD1 mice.

(**A**) Schematic of experimental design. Mice were treated with PDX for 30 min, followed by intraplantar injection of the TRPV1 agonist capsaicin (1 μg) and measurement of spontaneous pain. After the pain behavioral testing, Evans blue was injected intravenously to evaluate TRPV1-mediated neurogenic inflammation. (**B**) PDX blocks capsaicin-induced spontaneous pain, shown as reduced paw lifting or licking time within 5 min. (**C**) PDX blocks capsaicin-induced mechanical allodynia by von Frey test 10 min after injection. (**D**) Hind paw images showing capsaicin-induced edema 15 min after the Evans blue injection. (**E, F**) Quantification of paw edema by weight (E) and Evans blue concentration at OD 620 nM (F) in hind paws of mice treated with capsaicin with or without PDX (100 ng, i.v.). (**G**) DRG calcium images from four groups treated with PBS, PDX (30 nM), PBS + capsaicin (300 nM), and capsaicin + PDX. Scale bar, 100 μm. Note that Figure S12G is the same image as Figure 10H. (**H**) Heat map of DRG calcium signal in PBS + capsaicin and PDX + capsaicin groups. Each group consists of 51 DRG neurons. Scale bars, 3 min. (**I**) Percentage of capsaicin-responsive DRG neurons showing the effects of PDX pretreatment. (**J, K**) Co-localization of *Gpr37* with *Trpv1* in mouse DRG based on single-cell RNA-seq data. (J) UMAP plot showing *Gpr37* (blue) and *Trpv1* (red) expression. (K) Percentage of *Gpr37*/*Trpv1* co-expressing cells in mouse DRGs.

Data are represented as mean ± SEM and statistically analyzed by unpaired Student's t-test (B, E, F, I) and one-way ANOVA with Bonferroni's post hoc test (C). ***$P < 0.001$, ****$P < 0.0001$; $n = 6$ mice per group (3 males and 3 females), ▲ male, ● female.

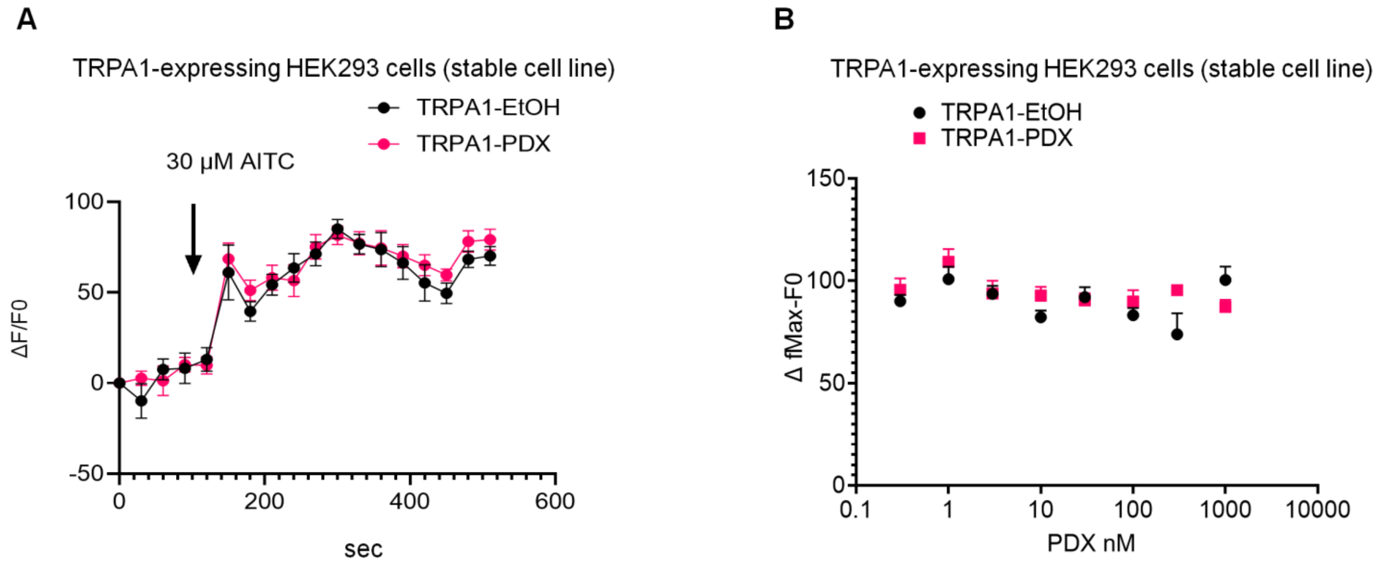

Supplemental Figure 13. PDX has no direct effects on TRPA1 activity in TRPA1-expressing HEK293 cells.

(**A**) Effects of 30 nM PDX on AITC-induced calcium influx in TRPA1-expressing HEK293 cells, visualized using Fluo-8 calcium imaging (n = 6 wells). (**B**) Dose–response curve of PDX effects (1 nM – 300 nM) on AITC (30 µM)-induced calcium influx in TRPA1-expressing HEK293 cells. Ethanol (EtOH) was used as vehicle control (n = 6 wells per condition).

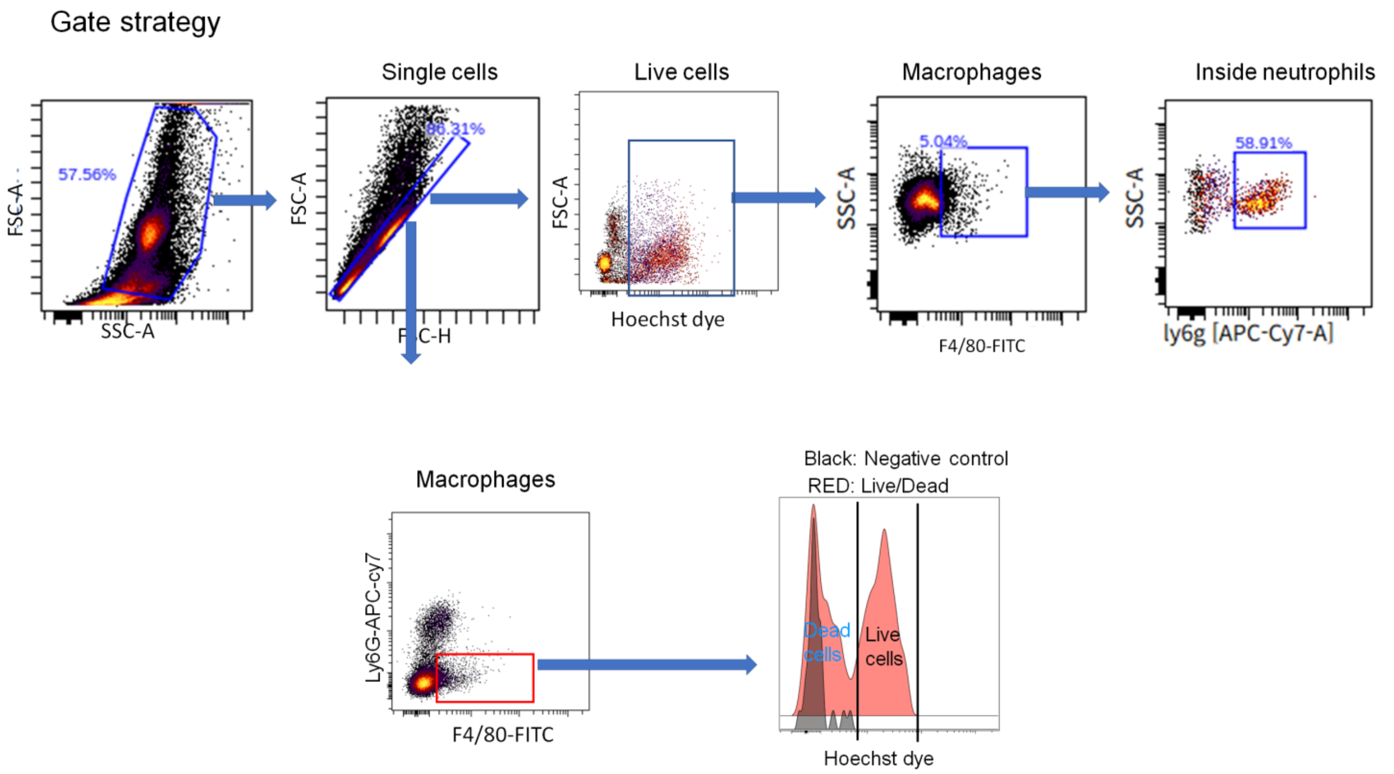

Supplemental Figure 14. Gating strategy for in vivo efferocytosis detection in muscles of bone fracture area using flow cytometry.

Supplemental Table 1: Numbers and sexes of animals used in this study

Supplemental Table 2: Differentially expressed genes in PDX-treated (Fracture + PDX) versus non-treated (Fracture + vehicle) groups from the DRG database of bulk RNA-sequencing.

Supplemental Table 3: Statistical details for all the figures presented in this study.

Supplemental Table 4: Lipidomic analysis in muscle near bone fracture of CD1 mice. Mice were given either sham surgery or tibial bone fracture surgery, and muscle was collected 3 days after in PBS. Lipid mediator quantification was carried out using liquid chromatography and tandem mass spectrometry (LC-MS/MS) on SCIEX Triple Quad 7500. Lipid mediators were identified by matching retention time and prominent ions in their MS-MS to those of authentic Serhan Lab Standards (see Methods section). n = 3 male mice per group. Results expressed as pg per muscle sample (3 mg tissue per muscle sample). § = not identified. (See below)

Table S4a. SPM profile of tibial muscle of mice with tibial bone fracture surgery

		(pg/ 3 mg sample)*		
		Fracture 1	Fracture 2	Fracture 3
	RvD1	§	§	§
	17R-RvD1	§	§	§
	RvD2	§	§	§
	17R-RvD2	§	§	§
	RvD3	§	§	§
	17R-RvD3	§	§	§
	RvD4	§	§	§
	RvD5	10.5	101.0	15.6
	PD1	2.7	5.5	§
	PDX	123.3	33.5	145.3
	MaR1	§	§	§
	MaR2	16.0	38.1	30.0
	17-HDHA	587.2	1,793.5	696.2
	14-HDHA	4,163.8	1,2471.3	7,083.8
Pro-resolving	RvE1	§	§	§
	RvE2	§	§	§
	RvE4	§	§	§
	18-HEPE	19.5	36.8	20.6
	15-HEPE	332.0	1,235.7	249.2
	12-HEPE	7,527.4	21,788.7	16,129.0
	5-HEPE	88.5	556.3	50.9
	LXA4	§	§	§
	AT-LXA4	§	§	§
	LXB4	§	§	§
	5S,15S-DiHETE	§	§	§
	15-HETE	1,724.8	3,708.7	2,530.0
Pro-inflammatory	LTB_4 (+5S,12S-DiHETE)	56.2	1,232.4	175.0
	6E-LTB_4	82.4	496.7	141.7

6E,12S-LTB_4	107.2	701.2	206.1
5S,6R-DiHETE	3.4	12.0	4.9
5S,6S-DiHETE	24.1	163.2	58.8
5-HETE	743.4	4,867.1	845.6
PGD2	743.9	1,194.8	935.8
PGE2	1,149.6	1,767.9	1,973.3
12-HETE	27,369.3	55,594.7	49,166.5
TxB2	769.9	2,107.2	2,024.5

§ = not identified.

Table S4b. SPM profile of tibial muscle of mice with sham surgery

		(pg/3 mg sample)*		
		Sham 1	Sham 2	Sham3
Pro-resolving	RvD1	§	§	§
	17R-RvD1	§	§	§
	RvD2	§	§	§
	17R-RvD2	§	§	§
	RvD3	§	§	§
	17R-RvD3	§	§	§
	RvD4	§	§	§
	RvD5	8.6	28.8	39.8
	PD1	§	8.1	13.5
	PDX	58.9	15.8	103.0
	MaR1	§	§	§
	MaR2	7.1	30.2	37.8
	17-HDHA	230.5	1,332.6	1,613.5
	14-HDHA	650.0	4,272.2	6,370.7
	RvE1	§	§	§
	RvE2	§	§	§
	RvE4	§	§	§
	18-HEPE	5.9	10.0	8.4
	15-HEPE	72.6	493.0	654.2
	12-HEPE	818.7	2,641.7	4,855.3
	5-HEPE	29.3	57.6	67.8
	LXA_4	§	§	§
	AT-LXA_4	§	§	§
	LXB_4	§	§	§
	5S,15S-DiHETE	§	§	§
	15-HETE	713.6	2,251.7	2,853.4
Pro-inflammatory	LTB_4 (+5S,12S-DiHETE)	23.5	41.9	85.6

6E-LTB_4	29.6	91.4	175.2
6E,12S-LTB_4	39.8	104.9	176.5
5S,6R-DiHETE	§	1.5	§
5S,6S-DiHETE	13.5	21.0	28.3
5-HETE	302.0	616.7	858.6
PGD2	357.1	368.1	516.3
PGE2	818.1	1,173.9	814.3
12-HETE	6,567.9	16,273.5	2,7503.9
TxB2	295.1	730.4	558.5

§ = not identified.

*Table I Legend: C**D1 male mice were given either sham surgery or tibial bone fracture surgery,** Surrounding muscle was collected 3 days after in PBS. Lipid mediator identification and quantitation were carried out using liquid chromatography and tandem mass spectrometry (LC-MS/MS) on SCIEX Triple Quad 7500. Lipid mediators were identified by matching retention and prominent ions in their MS-MS to those of authentic Serhan Lab Standards (see Methods section). Results expressed as pg/tibial sample. § = not identified.

Supplemental Table 5: Lipidomic analysis in the spleen of CD1 mice. Mice were given either sham surgery or tibial bone fracture surgery, and the spleen was collected 3 days after in PBS. Lipid mediator quantification was carried out using liquid chromatography and tandem mass spectrometry (LC-MS/MS) on SCIEX Triple Quad 7500. Lipid mediators were identified by matching retention time and prominent ions in their MS-MS to those of authentic Serhan Lab Standards (see Methods section). n = 3 male mice per group. Results expressed as pg/60 mg spleen tissue. § = not identified. (See below)

Table S5a. SPM profile of spleens of mice with tibial fracture surgery

		(pg/60 mg tissue) *.		
		Fracture 1	Fracture 2	Fracture 3
Pro-resolving	RvD1	127.8	118.7	99.5
	17R-RvD1	§	§	§
	RvD2	4.1	2.8	4.6
	17R-RvD2	§	§	§
	RvD3	§	§	§
	17R-RvD3	§	§	§
	RvD4	§	§	§
	RvD5	79.8	63.7	93.6
	PD1	§	§	§
	PDX	228.7	100.8	147.8
	MaR1	§	§	§
	MaR2	159.1	141.8	240.7
	17-HDHA	1,501.7	954.6	1,168.7
	14-HDHA	32,125.1	27,526.5	31,606.6
	RvE1	§	§	§
	RvE2	§	§	§
	RvE4	39.7	35.1	42.2
	18-HEPE	224.0	201.4	229.5
	15-HEPE	891.6	371.8	489.3
	12-HEPE	55,251.0	50,283.3	55,627.8
	5-HEPE	84.0	104.2	132.2
	LXA_4	§	§	§
	AT-LXA_4	§	§	§
	LXB_4	§	§	§
	5S,15S-DiHETE	2,771.9	2,314.5	3,256.0
	15-HETE	14,128.0	10,336.5	13,619.8
Pro-inflammatory	LTB4 (+5S,12S-DiHETE)	942.1	1,132.9	1,941.6
	6E-LTB4	638.3	399.8	752.8

6E,12S-LTB_4	1,111.7	789.6	1,333.9
5S,6R-DiHETE	5.2	2.6	7.3
5S,6S-DiHETE	13.8	18.4	25.4
5-HETE	339.5	506.8	654.3
PGD2	55,048.1	47,814.0	102,755.2
PGE2	§	42,768.9	§
12-HETE	98,279.9	80,811.5	89,452.5
TxB2	187,708.5	230,452.2	465,035.8

§ = not identified.

Table S5b. SPM profile of spleens of mice with sham surgery

		(pg/60 mg tissue)*		
		Sham 1	Sham 2	Sham 3
	RvD1	80.4	165.2	105.8
	17R-RvD1	§	§	§
	RvD2	6.4	2.0	4.3
	17R-RvD2	§	§	§
	RvD3	§	§	§
	17R-RvD3	§	§	§
	RvD4	§	§	§
	RvD5	139.6	62.8	81.2
	PD1	§	§	§
	PDX	171.8	76.9	150.7
	MaR1	§	§	§
	MaR2	129.4	92.7	150.4
	17-HDHA	2,192.6	995.2	1,782.8
	14-HDHA	11,036.8	23,325.4	20,961.3
Pro-resolving	RvE1	§	§	§
	RvE2	§	§	§
	RvE4	114.5	46.4	43.9
	18-HEPE	180.0	227.6	211.8
	15-HEPE	1,608.3	500.1	844.1
	12-HEPE	26,653.0	46,583.5	41,874.8
	5-HEPE	626.5	251.3	157.1
	LXA4	§	§	§
	AT-LXA4	§	§	§
	LXB4	§	§	§
	5S,15S-DiHETE	2,484.1	2,203.2	1,950.2
	15-HETE	20,855.4	14,267.7	17,029.4
Pro-inflammatory	LTB_4 (+5S,12S-DiHETE)	1,189.8	1,757.3	910.3
	6E-LTB_4	305.3	382.4	465.5
	6E,12S-LTB_4	412.1	623.8	908.2

5S,6R-DiHETE	5.2	5.0	3.3
5S,6S-DiHETE	53.9	29.5	23.1
5-HETE	3,298.3	1,387.7	868.9
PGD2	42,586.4	49,026.2	49,435.4
PGE2	44,523.1	§	38,570.3
12-HETE	67,991.0	83,355.6	85,447.8
TxB2	207,245.3	335,118.1	169,090.0

§ = not identified.

*Table I Legend: **CD1 male mice were given either sham surgery or tibial bone fracture surgery**, Spleens were collected 3 days after in PBS. Lipid mediator quantitation was carried out using liquid chromatography and tandem mass spectrometry (LC-MS/MS) on SCIEX Triple Quad 7500. Lipid mediators were identified by matching retention time and prominent ions in their MS-MS to those of original authentic Serhan Lab Standards (see Methods section). Results expressed as pg/60 mg spleen tissue. § = not identified.

Supporting Data Values: Raw data in a single excel file.

Supplemental Video 1: A 60-minute video demonstrates delayed calcium flux (green) and zymosan particle phagocytosis (red) in peritoneal macrophages after PBS treatment.

Supplemental Video 2: A 60-min video demonstrates transient calcium flux (green) and accelerated, enhanced phagocytosis of zymosan particles (red) in peritoneal macrophages after PDX treatment.

Supplemental File 1: BioRender publication license for all the images and drawings.

Supplemental Materials and Methods

Cell cultures of HEK293T cells, DRG cells, pM*φ*s, and THP1 cells

HEK293T cell culture and transfection. The HEK293T flip-in cell line was purchased from the Duke Cell Culture Facility. The HEK293-hTRPA1 stable cell line was purchased from SB Drug Discovery (Cat # SB-HEK-TRPA1). Cells were cultured in Dulbecco's Modified Eagle's Medium containing 10% (v/v) FBS (Gibco, Thermo Fisher Scientific). Transfection (2 μg cDNA) was performed with Lonza electroporation at 70% confluency, and the transfected cells were cultured in the same growth medium for 48 h before use. The hGPR37-V5 pLenti304 plasmids were obtained from the DNASU Plasmid Repository.

Primary pMφ culture. pM*φ*s were collected by peritoneal lavage with 5 ml warm PBS containing 1 mM EDTA. Cells were incubated in DMEM supplemented with 10% FBS at 37°C for 1 hour in a Petri dish and washed with PBS to eliminate nonadherent cells. The adherent cells were used as pM*φ*s. pM*φ*s were used after 2-3 days of culture.

THP1 cell culture. THP1 cell line was purchased from the Duke Cell Culture Facility. Cells were cultured in RPMI-1640 Medium containing 10% (v/v) FBS (Gibco, Thermo Fisher Scientific) and 0.05 mM 2-mercaptoethanol. To induce THP1 monocyte differentiation to macrophages, 25 nM of phorbol 12-myristate 13-acetate (PMA) was added to cultures.

In vitro calcium imaging in HEK293T cells and pM*φ*s

HEK293T cells were loaded with 5 μM Fura2-AM (Invitrogen, Thermo Fisher Scientific, F1221) for 45 min and then resuspended in normal external buffer (140 mM NaCl, 5 mM KCl, 2 mM CaCl2, 2 mM MgCl2, 10 mM HEPES, titrated to pH 7.4 with NaOH) or external Ca^{2+}-free buffer (140 mM NaCl, 2 mM MgCl2, 5 mM EGTA, 10 mM HEPES, titrated to pH 7.4 with NaOH). pM*φ*s collected from WT and *Gpr37*$^{-/-}$ mice and TRPA1 HEK293 cells were loaded with 3 μM Fluo8-AM (AAT Bioquest, 21055) for 30 min and then resuspended in DMEM medium. Images of HEK293T cells with an excitation wavelength of 340 nm and 380 nm or pM*φ*s with an excitation wavelength of 488 nm were captured with a cooled Digital CMOS camera (ORCA-Flash 4.0, Hamamatsu Photonics). The ratio of fluorescence intensity of the two wavelengths in each experiment, as the peak amplitude/intensity in the first 10 min after treatment, was analyzed using MetaFluor software (Molecular Devices). The Shutter speed and wavelength were controlled by the pe-300 Fura system (Cool LED). Values from each experiment were normalized to the baseline ratio of 340:380 nm or the basal

intensity for pMφs.

***Ex vivo* calcium imaging in whole-mount DRG from AAV-MaCPNS.2-hSyn-Gcamp6f-infected mice**

AAV-MaCPNS.2-hSyn-Gcamp6f was intraperitoneally (i.p.) administered to C57BL6 mice (3×10^{11} vg /mouse) on postnatal day 1 (P1). Four weeks after the AAV injection, L3-L5 DRG were collected for an ex vivo DRG calcium imaging experiment. Mice were anesthetized through i.p. injection of 1.5 g/kg urethane, and their body temperature was maintained at approximately 37°C using a heat pad throughout the procedure. DRG were harvested and kept in ACSF. Stable imaging was achieved by placing a mash on the DRG to fix the position of DRG. Live imaging was facilitated using a Zeiss 780 upright confocal microscope with a 20X water objective lens, with the focal plane depth ranging from 50 to 70 μm. Seven images were captured per cycle, and 50 cycles were recorded. Following the acquisition of baseline measurements for 3 min, drugs (diluted in ACSF) were added to dishes, and a total of 15 min or 25 min time-lapse recordings were performed for each DRG. The data were analyzed using FIJI software.

Dot blot assay for lipid-protein binding

Lipid membrane coating and protein overlay assay were conducted as previously described (6). Ethanol and chloroform-soluble fatty acids were directly loaded onto a hydrophobic PVDF membrane (MilliporeSigma). The membrane was coated with PDX and NPD1. The fatty acid–coated membranes were dried and blocked with 1% BSA. To express and isolate GPR37, HEK293 cells were transfected with GPR37 cDNA with a V5 tag (GPR37-V5) or with an empty vector (Mock transfection) using Lipofectamine 3000 (Invitrogen, Thermo Fisher Scientific). Cell lysates were incubated overnight with the coating membrane at 4°C, and the binding was detected by anti-V5–tagged antibody (mouse, 1:1,000, Thermo Fisher Scientific, catalog 46-0705). The blots were further incubated with an HRP-conjugated secondary antibody and developed in ECL solution (Pierce, Thermo Fisher Scientific). The intensity of lipid-protein binding was evaluated using ImageJ software.

ELISA

Mouse ELISA kits for IL-1β, TNF-α, and IL-10 were purchased from R&D Systems (catalog MLB00C for IL-1β, catalog MTA00B-1 for TNF-α, and catalog M1000B for IL-10). ELISA was performed using culture media of pMφs. For each ELISA assay, 50 μl of culture medium was used. ELISA was conducted according to the manufacturer's instructions. The standard curve was included in each experiment.

Quantitative real-time RT-PCR

Total RNA from the cultures was extracted using the Direct-zol RNA MiniPrep Kit (Zymo Research), and 0.5–1 μg RNA was reverse transcribed using the iScript cDNA Synthesis Kit (Bio-Rad). Specific primers, including the GAPDH control, were designed using IDT SciTools Real-Time PCR software. We performed gene-specific mRNA analyses using the Bio-Rad CFX96 system. Quantitative PCR amplification reactions contained the same amount of reverse transcription product, including 7.5 μl of 2× iQSYBR Green Mix (Bio-Rad) and 100–300 nM forward and reverse primers in a final volume of 15 μl. The primer sequences are listed below. Primer efficiency was obtained from the standard curve and integrated for the calculation of relative gene expression, which was based on real-time PCR threshold values of different transcripts. The primer sequences (5' to 3') are as follows:

Il-1β: forward - TTGTGGCTGTGGAGAAGCTGT, reverse - AACGTCACACACCAGCAGGTT;

Tnf-α: forward - AGAAGTTCCCAAATGGCCTCCCT, reverse - TACAACCCATCGGCTGGCACCAC;

Il-10: forward - TGTCAAATTCATTCATGGCCT, reverse - ATCGATTTCTCCCCTGTGAA;

Gapdh : forward - AGGTCGGTGTGAACGGATTTG, reverse - GGGGTCGTTGATGGCAACA.

Bulk RNA sequencing and pathway analysis of DRG tissues

Total RNA was extracted from L3-L5 DRG of mice with sham surgery and fracture surgery treated with vehicle or PDX mice using RNeasy® Mini Kit (QIAGEN, Cat# 74104). RNA quantity and quality were assessed with a NanoDrop™ One spectrophotometer (Thermo Fisher Scientific). All samples displayed a 260:280 ratio greater than 2.0 and RNA Integrity Numbers (RINs) above 8.0, with RNA concentration > 150 ng/μL and the total RNA yield > 3 μg/sample. The Poly(A) RNA sequencing library was prepared according to Illumina's TruSeq-stranded-mRNA sample preparation protocol. RNA integrity was verified using the Agilent Technologies 2100 Bioanalyzer. Poly(A) tail-containing mRNAs were purified using oligo-(dT) magnetic beads, undergoing two rounds of purification. After purification, the poly(A) RNA was fragmented in a divalent cation buffer at elevated temperature, and the DNA library was then constructed. Quality control and quantification of the sequencing library were conducted using the Agilent Technologies 2100 Bioanalyzer High Sensitivity DNA Chip. Paired-end sequencing was carried out on the Illumina NovaSeq 6000 sequencing system by LC Sciences.

RNA-seq differential gene expression data were analyzed using QIAGEN's Ingenuity Pathway Analysis (IPA, QIAGEN Redwood City, www.qiagen.com/ingenuity) software version 01-16. Top Canonical Pathway analysis was conducted to compare PDX and vehicle treated groups with fracture. The significance of the

association between the dataset and canonical pathways was measured in two ways: 1) A ratio of the number of molecules from the dataset that map to the pathway, divided by the total number of molecules that map to the canonical pathway. 2) Fisher's exact test was used to calculate a p-value determining the probability that the association between the genes in the dataset and the canonical pathway is explained by chance alone. Volcano Plot: The volcano plot visualizes the relationship between the statistical significance (-log10 p-value) and the magnitude of change (log2 fold change) for genes in the study. Genes related to the pathways of interest are labeled on the plot (Supplemental Table 2).

In vivo recordings of C-fiber reflex using electromyogram

As previously described, an electromyogram (EMG) was used to record the C-fiber reflexes from the biceps femoris (7). Briefly, mice were anesthetized with 1.5% isoflurane, and needle electrodes were inserted subcutaneously into the medial part of the 3rd and 4th toes. The electrodes delivered electrical stimuli with single square waves of 1 ms from a constant current isolated stimulator (DS3, Digitimer Ltd, London, England) to evoke C-fiber reflexes. EMG signals were recorded using a pair of platinum-iridium electrodes inserted into the left biceps femoris muscle. These signals were amplified via a microelectrode amplifier (A-M system, Sequim, WA), and they were then recorded using an acquisition system (Digidata 1440, Molecular Devices). The C-fiber reflex threshold (Tc) was identified when the EMG signals corresponding to C-fiber activities were elicited. After detecting Tc, the EMG reflex resulting from stimulation at 2-fold Tc was established as the noxious stimulation-induced responses. C-fiber reflexes were recorded before and after 30 min PDX treatment (peri-sciatic nerve injection， 20 ng in 20 μL; intraperitoneal injection, 150 ng in 100 μL), with PBS as vehicle control.

Computer simulations

The protein sequence of human GPR37 (hGPR37) was downloaded from the UniProt database (ID: O15354) in fasta format. Homology modeling was performed using the GPCR-ModSim server, which employs an automated approach for template selection and model generation (8). To elucidate the binding mode of all ligands in the binding site of the homology model of hGPR37, docking studies were performed with the help of Autodock4 software (9). Before the docking, the hGPR37 structure was prepared using the AutoDock Tools 4 software, which involved adding hydrogen atoms, assigning Gasteiger charges, and defining rotatable bonds. Based on previous mutagenesis studies and conservation analysis, the docking grid was centered on the putative binding site, encompassing residues identified as crucial for ligand interaction. For each ligand, 20

docking runs were performed, and the top-scoring poses were selected for further analysis. Molecular dynamics simulation and docking grid analysis were performed as previously described (10). The 100 ns of molecular dynamics simulation (MDS) was performed on the cluster 1 structure to assess the stability of the docking complex.

Plasmids and AAV production

pAdDeltaF6 was a gift from James M. Wilson (Addgene plasmid #112867). pUCmini-iCAP-AAV.MaCPNS2 was a gift from Viviana Gradinaru (Addgene plasmid # 185137). pAAV.CAG.GCaMP6f.WPRE.SV40 (Addgene plasmid #100836) and pAAV.Syn.GCaMP6f.WPRE.SV40 (Addgene plasmid # 100837) were gifts from Douglas Kim & GENIE Project. HEK293T cells were cultured in DMEM supplemented with 10% fetal bovine and 1.5 x10^7 cells were seeded per 15 cm dish 24 h before transfection. To produce each virus, a total of six dishes were prepared. Cells were then transfected with 30 µg pAd-DELTA F6, 15 µg serotype plasmid AAV.MaCPNS2, and 15 µg AAV plasmid with PEI MAX (Polysciences, Cat#24765). After 72 h, cells were harvested and lysed in 4 ml of lysis buffer (15 mM NaCl, 5 mM Tris-HCl, pH 8.5) using three freeze-thaw cycles, and incubated with 50 U/ml Benzonase (Millipore, Cat#70664) for 30 min at 37°C. After spinning down at 4500 rpm for 30 min at 4°C, the supernatant was added to the top of 15%, 25%, 40% and 60% stacked iodixanol gradients and centrifuged using a Beckman Ti-70 rotor, spun at 67,000 rpm for 1.5 h at 18°C. The viral solution was then collected from the interface between 40%-60% iodixanol gradients, washed with 1X PBS (Gibco, Cat#14190144), and concentrated with a 100 kDa filter (Millipore, Cat#UFC910008). The purified virus was aliquoted and stored at -80°C until use.

Paw edema assessment and Evans blue test

The weight of mouse hind paw was used to determine paw swelling (edema) after capsaicin and AITC injection. To assess vascular permeability, which is associated with paw swelling, Evans blue dye (1%, 200 µl) was injected into the tail veins of all mice 15 minutes after the capsaicin and AITC injections (11). Photographs of the hind paws were taken 15 minutes post-injection. The paws were then placed in a 65°C oven for 48 hours to remove moisture content and ensure consistent weight measurements. Subsequently, the dried paw tissues were weighed, crushed, and soaked in 500 µl of formamide. The samples were heated in a water bath at 55°C for 24–48 hours to extract the Evans blue dye. The formamide/Evans blue mixture was centrifuged at 4000 × g for 10 minutes, and the Evans blue dye concentration in the supernatant was quantified by measuring absorbance at 630 nm using a microplate reader.

Flow Cytometry Analysis of Efferocytosis in Bone Fracture Tissue

Wild-type (WT) and *Gpr37* knockout (KO) mice were subjected to bone fracture, and muscle tissue surrounding the fracture site was harvested 3 days post-injury. Tissues were minced and enzymatically digested in 1 mg/mL collagenase/Dispase (Roche) for 60 minutes at 37°C with gentle agitation. Following digestion, samples were filtered through a 50 µm cell strainer to obtain single-cell suspensions. Enzymatic activity was quenched using 10% fetal bovine serum (FBS), and cells were washed with PBS containing 10 mM EDTA. Cells were then resuspended and incubated for 10 minutes with Invitrogen's permeabilization solution to allow intracellular staining. To block Fc receptors, cells were incubated with anti-CD16/32 antibody (1 µg/ml) in 10% bovine serum albumin (BSA) for 1 hour at 4°C. Cells were then stained with the following antibodies diluted 1:200 in cold PBS + 10% BSA + 10 mM EDTA: F4/80-FITC(Biolegend: 1:200) for macrophage identification, Ly6G-APC-Cy7(Biolegend, 1:200) for neutrophil detection**,** Hoechst dye **(Sigma)** for live/dead cell discrimination, and nuclear staining. After staining, cells were washed in PBS with EDTA. The flow cytometry events were acquired in a BD FACS Canto II flow cytometer by using BD FACS Diva 8 software (BD Bioscience). Data were analyzed using Cytobank Software (https://www.cytobank.org/cytobank). The gating strategy was illustrated in Supplemental Figure 14.

Spatial transcriptomic analysis in human DRG

Human dorsal root ganglia (DRG) were obtained from two donors (a 63-year-old male and a 63-year-old female) through the National Disease Research Interchange (NDRI) with exemption permission from the Duke University Institutional Review Board (IRB). Postmortem L3–L4 DRG were delivered in ice-cold cell culture medium within 28 hours of death. Upon receipt, tissues were immediately dissected and fixed in 4% paraformaldehyde followed by cryoprotection in 30% sucrose at 4°C for a minimum of three nights.

Visium HD Experiment. OCT-embedded frozen DRG tissues were cryosectioned at 20 µm thickness and stored at –80°C. Slides were rehydrated, H&E stained, and destained following the 10x Genomics Visium CytAssist Spatial Gene Expression for Fixed Frozen – Rehydration, H&E Staining, Imaging & Decrosslinking protocol (CG000662). Brightfield imaging was performed using a Zeiss Axioscan Z1 slide scanner with a 20× objective. Following imaging, tissue sections were decrosslinked according to the *Visium HD FFPE Tissue Preparation Handbook* (CG000684) and then processed using the *Visium HD Spatial Gene Expression Reagent Kits* (CG000686).

In brief, whole transcriptome probe panels were applied to each section for hybridization and ligation across the transcriptome. Slides were loaded into the Visium CytAssist instrument, which enabled alignment

of the tissue section with the Visium HD slide. Gene expression probes and antibody tags were released through CytAssist-enabled RNA digestion and tissue removal, allowing capture by spatially barcoded oligonucleotides on the slide surface. The slides were then removed for extension and downstream library preparation. Barcoded ligation products were amplified, purified, and indexed by PCR for multiplexing, with the optimal number of cycles determined by qPCR. Final libraries were quantified using KAPA qPCR and sequenced on an Illumina NovaSeq X Plus 1.5B flow cell with the following read lengths: Read 1: 43 cycles; i7 Index: 10 cycles; i5 Index: 10 cycles; Read 2: 50 cycles.

Data Processing and Analysis: Raw FASTQ files were processed using Space Ranger software (v3.1.2, 10x Genomics) for alignment to the reference transcriptome and generation of gene expression matrices at 2 µm, 8 µm, and 16 µm binning resolutions. CytAssist images were manually aligned to high-resolution H&E images using the Loupe Browser (10x Genomics) prior to running the spaceranger count command. Inputs included the CytAssist image, microscope image, FASTQ files, probe set, reference transcriptome, and alignment JSON file. Outputs included spatially resolved gene expression matrices and annotated tissue images.

Supplemental references

1. Serhan CN. Pro-resolving lipid mediators are leads for resolution physiology. *Nature.* 2014;510(7503):92-101.
2. Ji RR. Specialized Pro-Resolving Mediators as Resolution Pharmacology for the Control of Pain and Itch. *Annu Rev Pharmacol Toxicol.* 2023;63:273-93.
3. Bang S, Jiang C, Xu J, Chandra S, McGinnis A, Luo X, et al. Satellite glial GPR37L1 and its ligand maresin 1 regulate potassium channel signaling and pain homeostasis. *J Clin Invest.* 2024;134(9).
4. Chiang N, Libreros S, Norris PC, de la Rosa X, and Serhan CN. Maresin 1 activates LGR6 receptor promoting phagocyte immunoresolvent functions. *J Clin Invest.* 2019;129(12):5294-311.
5. Jin H, Zhang C, Zwahlen M, von Feilitzen K, Karlsson M, Shi M, et al. Systematic transcriptional analysis of human cell lines for gene expression landscape and tumor representation. *Nat Commun.* 2023;14(1):5417.
6. Bang S, Xie YK, Zhang ZJ, Wang Z, Xu ZZ, and Ji RR. GPR37 regulates macrophage phagocytosis and resolution of inflammatory pain. *J Clin Invest.* 2018;128(8):3568-82.
7. Cui X, Wei W, Zhang Z, Liu K, Zhao T, Zhang J, et al. Caffeine Impaired Acupuncture Analgesia in Inflammatory Pain by Blocking Adenosine A1 Receptor. *J Pain.* 2024;25(4):1024-38.
8. Esguerra M, Siretskiy A, Bello X, Sallander J, and Gutierrez-de-Teran H. GPCR-ModSim: A comprehensive web based solution for modeling G-protein coupled receptors. *Nucleic Acids Res.* 2016;44(W1):W455-62.
9. Morris GM, Huey R, Lindstrom W, Sanner MF, Belew RK, Goodsell DS, et al. AutoDock4 and AutoDockTools4: Automated docking with selective receptor flexibility. *J Comput Chem.* 2009;30(16):2785-91.
10. Bang S, Donnelly CR, Luo X, Toro-Moreno M, Tao X, Wang Z, et al. Activation of GPR37 in

macrophages confers protection against infection-induced sepsis and pain-like behaviour in mice. *Nat Commun.* 2021;12(1):1704.

11. Zhang X, Retyunskiy V, Qiao S, Zhao Y, and Tzeng CM. Alloferon-1 ameliorates acute inflammatory responses in lambda-carrageenan-induced paw edema in mice. *Sci Rep.* 2022;12(1):16689.
