# Supplementary figures and images for: Protectin DX resolves fracture-induced postoperative pain in mice via neuronal signaling and GPR37-activated macrophage efferocytosis
Source: J Clin Invest. 2026 Jan 16;136(2):e190754. doi: 10.1172/JCI190754 (PMC12807480; doi:10.1172/JCI190754)

Figure 4H

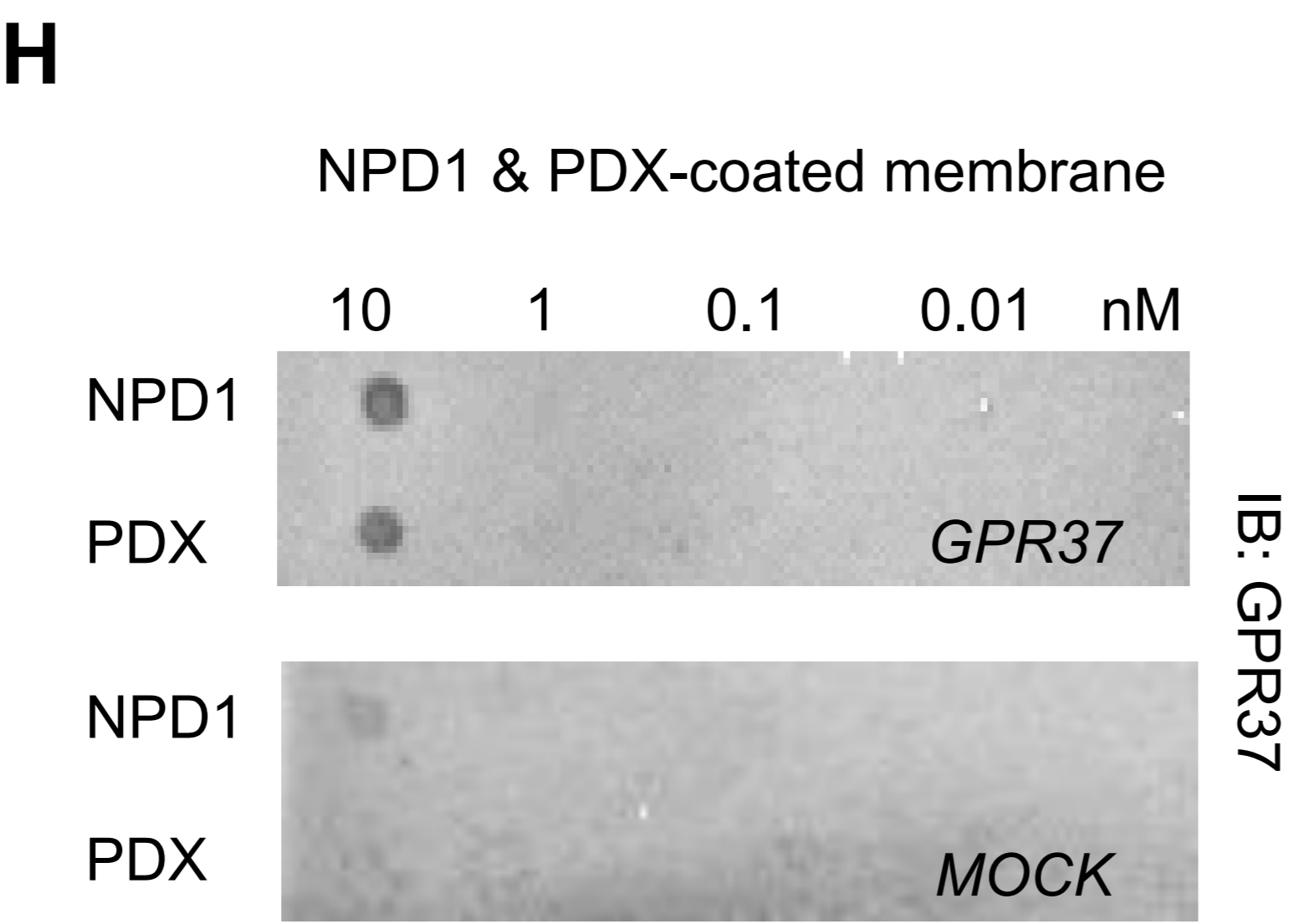

Supplement: Unedited blot and gel images [file jci-136-190754-s140.pdf]
